# Impact of pain, fatigue and bowel incontinence on the quality of life of people living with inflammatory bowel disease: A UK cross‐sectional survey

**DOI:** 10.1002/ueg2.12668

**Published:** 2024-10-19

**Authors:** Chris Roukas, Laura Miller, Fionn Cléirigh Büttner, Thomas Hamborg, Imogen Stagg, Alisa Hart, Vladimir Sergeevich Gordeev, James O. Lindsay, Christine Norton, Borislava Mihaylova

**Affiliations:** ^1^ Health Economics and Policy Research Unit Wolfson Institute of Population Health Queen Mary University of London London UK; ^2^ Pragmatic Clinical Trials Unit Wolfson Institute of Population Health Queen Mary University of London London UK; ^3^ Faculty of Nursing Midwifery and Palliative Care King's College London London UK; ^4^ IBD Unit St Mark's Hospital London UK; ^5^ Centre for Immunobiology Blizard Institute Faculty of Medicine and Dentistry Queen Mary University of London London UK; ^6^ Health Economics Research Centre Nuffield Department of Population Health University of Oxford Oxford UK

**Keywords:** bowel incontinence, Crohn's disease, fatigue, inflammatory bowel disease, pain, quality of life, ulcerative colitis

## Abstract

**Background and Aims:**

People with inflammatory bowel disease (IBD) often experience pain, fatigue and bowel incontinence and are at an increased risk of anxiety and depression. Our aim was to assess the impact of these symptoms on health‐related quality of life (QoL) in IBD.

**Methods:**

In the IBD‐BOOST survey, over 26,000 people with IBD across the UK were approached; 8486 participant‐completed surveys were returned. Participants' QoL was measured using the EQ‐5D‐5L questionnaire and their QoL was calculated on a scale ranging from 1 (perfect health) to −0.594 (worst health). Item non‐response was imputed. Stages of linear regression models assessed the associations of symptoms with QoL controlling for IBD type, socio‐demographic characteristics, co‐morbidities and, in further analysis, for IBD activity and IBD control.

**Results:**

The EQ‐5D‐5L questionnaire was fully completed by 8093 (95.4%) participants (mean age of 50 years [SD 15]; 49% with Crohn's disease). The mean QoL was 0.76 (SD 0.23). From the three IBD‐related symptoms, pain was associated with the largest QoL decrement (−0.159), followed by fatigue (−0.140) and bowel incontinence (−0.048). Co‐occurrence of pain and fatigue further reduced QoL. Clear graded associations were observed between symptom severity and QoL decrements (all trend *p* < 0.001). Depression and anxiety were also associated with significant QoL decrements (−0.102 and −0.110 for moderate‐to‐severe anxiety and moderately severe depression, respectively). Worse IBD control and higher IBD activity were associated with lower QoL.

**Conclusions:**

We report strong associations between symptoms of pain, fatigue, bowel incontinence, anxiety, depression, and their severity and reduced QoL in IBD. These estimates could inform future IBD management interventions.

## Graphical abstract

The study reports strong associations between symptoms of pain, fatigue, bowel incontinence, and their severity and reduced quality of life (QoL) in IBD. Pain is associated with the largest QoL reduction (−0.159), followed by fatigue (−0.140) and bowel incontinence (−0.048). Anxiety and depression further reduced QoL.

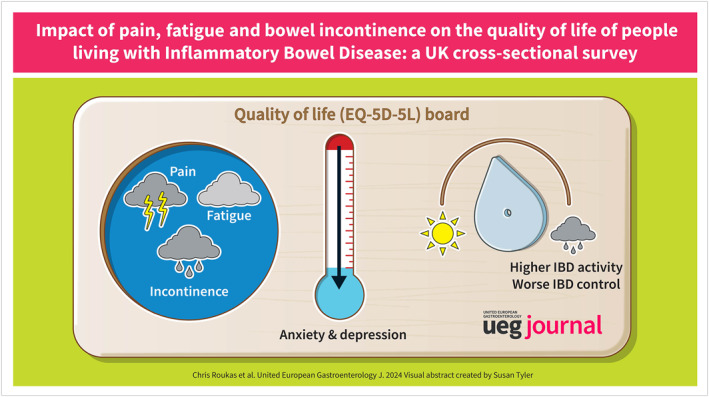

**Key summary**

**What is already known?**

- People with inflammatory bowel disease (IBD) experience pain, fatigue and bowel incontinence and are at increased risk of anxiety and depression. These symptoms limit peoples' quality of life (QoL) and ability to work and socialise.

**What are the significant and new findings**

- This is the first study to assess the relationship between these symptoms collectively and QoL in IBD. We report strong, severity‐graded associations between pain, fatigue, and bowel incontinence and reduced QoL in IBD. Depression and anxiety were also linked to significant QoL reductions.
- Our findings underline the need for interventions to improve the management of these common symptoms in people living with IBD.

## INTRODUCTION

Inflammatory Bowel Disease (IBD), predominantly comprising Crohn's disease (CD) and ulcerative colitis (UC), is a chronic condition causing inflammation in the gastrointestinal tract and is characterised by episodes of remission and relapse. It has been reported that in 2017 there were 6.8 million people diagnosed with IBD globally, 3 · 9 million women and nearly 3 · 0 million men.^1^

Disease onset typically occurs during young adulthood and treatment can be complex, including medical therapy with corticosteroids, 5‐aminosalicylic acid drugs, immunosuppressants, and biologics as well as surgery in some patients. People with IBD often experience urgency, an imperative and urgent need to defecate, which when severe or when toilet facilities are not available, can result in faecal incontinence, an involuntary loss of faeces. Many people living with IBD report bowel incontinence (75%), abdominal pain (62%), and fatigue (41%).^2^ They are also at increased risk of anxiety and depression compared to the general population.^3^ These symptoms can occur even when IBD is in remission and are associated with significantly reduced quality of life (QoL) as people living with IBD pursue employment, family planning, and personal achievements.^4^

Health‐related quality of life (HRQoL), a self‐reported measure of the functional impact of illness on individuals' daily life that is assessed using either a generic or disease‐specific questionnaire, is an important outcome in clinical trials.^5^ Generic HRQoL instruments have the advantage of enabling comparisons of effects across different diseases and are widely used in health economic and policy analyses. The five‐level version of the EuroQoL five‐dimensional (EQ‐5D‐5L) questionnaire^6^ is a frequently used generic HRQoL measure that asks respondents to indicate their health status on five dimensions: mobility, self‐care, usual activities, pain/discomfort, and anxiety/depression. EQ‐5D‐5L has been demonstrated to be feasible, consistent, and valid in patients with IBD.^7^

Previous studies have assessed the individual impact of symptoms of pain,^8^ fatigue,^9^ incontinence,^10^ and anxiety and depression^11^ on HRQoL in IBD using generic or disease‐specific measures. However, no study has yet assessed the relationship between pain, fatigue, bowel incontinence, anxiety, and depression collectively on QoL in IBD. To address this gap, data from a large UK cross‐sectional survey, the IBD‐BOOST survey, were used to quantify associations between symptoms of pain, fatigue, bowel incontinence, and the additional contribution of anxiety and depression on QoL in adults with IBD.

## MATERIALS AND METHODS

### IBD‐BOOST survey

The IBD‐BOOST survey was a multi‐site, cross‐sectional, participant‐completed survey in the UK that recruited adult participants with IBD from (i) hospital outpatient clinics, (ii) the UK IBD BioResource (www.ibdbioresource.nihr.ac.uk), and (iii) the Crohn's and Colitis UK charity (CCUK) (www.crohnsandcolitis.org.uk) between February 2019 and July 2022. Outpatient clinic patients were recruited via letter, email, or mobile phone text sent by clinical teams containing a link to the online survey. This link directed patients to an online participant information sheet, screening form, consent form, and the survey. Alternatively, patients received paper copies of these materials via post. The IBD BioResource had ethics committee permission to approach people who had indicated willingness to participate in research. Therefore, email invitations were sent to patients previously recruited to the IBD BioResource. CCUK approached their members by post or email. Participants with incomplete surveys received up to two reminders by text message, post, or email. Adults with IBD were also able to participate by following an online survey link posted on social media and selected IBD‐related websites.

The survey included 14 sections that collected information on participants' sociodemographic characteristics, disease history and health, current treatment for IBD, IBD control and disease activity levels, symptoms of pain, fatigue and bowel incontinence, anxiety and depression, and HRQoL.

### Measurement of HRQoL in the IBD‐BOOST survey

The HRQoL of IBD‐BOOST survey participants was measured using the EQ‐5D‐5L questionnaire. Participants were asked to rate their health across five domains—mobility, self‐care, usual activities, pain/discomfort, and anxiety/depression—by selecting one of five possible levels: ‘no problems’, ‘slight problems’, ‘moderate problems’, ‘severe problems’ or ‘extreme problems’. Using the UK National Institute for Health and Care Excellence recommended scoring algorithm, these problems were mapped onto EQ‐5D utility values with 1 representing a perfect health state, 0 representing a health state equivalent to death, and values less than 0 representing health states worse than death.^12^

### Pain, fatigue and bowel incontinence measures

The Patient‐Reported Outcomes Measurement Information System (PROMIS) (www.promishealth.org) questionnaires were used to measure patient‐reported symptoms of pain, fatigue and bowel incontinence in the past 7 days. In this study, we used the three‐item PROMIS Pain Intensity v1.0, the seven‐item PROMIS Fatigue Short Form v1.0, and the four‐item PROMIS Gastrointestinal Bowel Incontinence v1.0. Raw scores for each symptom scale range from 7 to 35, 3 to 15, and 4 to 20, respectively, with higher scores representing worse symptoms. PROMIS measures have demonstrated reliability, precision, and construct validity.^13^

Prior to analysis, PROMIS pain and PROMIS fatigue scores were converted to standardised T‐scores and then dichotomised, with a score of 60 or above indicating the presence of the symptom. PROMIS bowel incontinence scores of 5 or above indicated bowel incontinence.

### Anxiety and depression measures

To measure anxiety and depression, the Generalised Anxiety Disorder (GAD)‐7^14^ and the Patient Health Questionnaire (PHQ)‐9^15^ were administered. GAD‐7 scores ranged from 0 to 21 with scores of 0–4, 5–9, 10–14, and 15–21 indicating no anxiety, mild anxiety, moderate anxiety, and severe anxiety, respectively. Prior to analysis, moderate and severe scores^10^, ^11^, ^12^, ^13^, ^14^, ^15^, ^16^, ^17^, ^18^, ^19^, ^20^, ^21^ were combined into a moderate‐to‐severe anxiety category. PHQ‐9 scores ranged from 0 to 27 (with a score of 0–4 indicating no depression and higher scores corresponding to more severe depression). For analysis, scores of 0–4, 5–9, and 10 or greater were categorised as no depression, mild‐to‐moderate, and moderately severe depression, respectively. Both the GAD‐7 and PHQ‐9 have demonstrated excellent reliability, construct and criterion validity, and sensitivity to change.^16^, ^17^

### IBD activity and control

Disease activity of participants diagnosed with CD or UC was measured using a practical and non‐invasive tool, the two‐item Patient Reported Outcome PRO‐2.^18^, ^19^ PRO‐2 for CD is comprised of two items: abdominal pain and stool frequency, whereas PRO‐2 for UC is comprised of rectal bleeding and stool frequency. A higher score indicates worse levels of IBD activity. Similarly, IBD control was measured using the validated IBD‐Control questionnaire,^20^ comprising 13 items in addition to a 0–100 visual analogue scale (VAS). IBD control scores ranged from 0 to 16 (with lower scores representing worse disease control) and tertiles (1: 15–16 [best control], 2: 9–14 and 3: 0–8 [worst control]) were used in the analysis.

### Statistical methods

#### Missing data

Multivariable multivariate imputation by chained equations was performed to replace missing responses in returned questionnaires assuming that participants' missing responses for the EQ‐5D‐5L questionnaire, symptoms, and other measures were missing at random.^22^ Missing EQ‐5D‐5L questionnaire responses were imputed by predictive mean matching after all covariates, including the VAS score, were included in the imputation model. Missing values of other measures included in the model were imputed using the same approach. We imputed 10 datasets replacing missing data, performed statistical analyses (described below) on each dataset, and combined their estimates using Rubin's rule.^23^

#### Statistical modelling of QoL

We used regression modelling to estimate the strength of associations between key exposures and EQ‐5D‐5L QoL utility. The EQ‐5D utility data have an upper limit of 1 and are typically negatively skewed, with a high proportion of participants reporting full health (utility value of 1). Although different types of regression models have been proposed to accommodate these distributional features, ordinary least squares linear regression has remained the preferred model, both in terms of parsimony and because it minimises bias.^21^

Our approach to modelling QoL involved three pre‐specified stages of linear regression model development. All stages included the following core patient characteristics—IBD type (CD, UC, or other type of IBD), previous IBD surgery, and use of biological medications as binary variables, sociodemographic characteristics (age, gender, smoking status, body mass index [BMI]), clinical characteristics (physical and mental health comorbidities), and nationwide quintiles of index of multiple deprivation (IMD), a measure of socioeconomic deprivation in the UK^24^ (from quintile 1, the most deprived, to quintile 5, the least deprived) (Table S1).

Initially, a model was fitted including only IBD disease characteristics (IBD type, prior IBD surgery and use of biological medication) and confounders (age, gender, smoking status, BMI, IMD, and physical and mental comorbidities). Additional variables (time since diagnosis, pregnancy, education, employment, and relationship status) were retained only if statistically significantly associated with QoL (*p* < 0.05). At stage 1, IBD‐related symptoms (pain, fatigue and bowel incontinence) were included (dichotomised or categorised by severity) as key exposures alongside the covariates and confounders retained in the initial model. At stage 2, interactions between the dichotomised IBD‐related symptoms with more than 20% overlap were included and retained only if statistically significant. At stage 3, anxiety and depression symptoms were included as main effects to investigate their independent association with QoL, and with further interaction terms to examine whether they modify the relationship between IBD‐related symptoms and QoL. This model development strategy is summarised in Table S2.

### Sensitivity analyses

We assessed the effect of the addition of adjustments for IBD activity and IBD control in the models on the estimated associations between symptoms and QoL. In a further analyses, the associations were estimated (a) separately among participants with CD and among participants with UC or other IBD, and (b) separately among women and men.

All analyses were performed using Stata v.17.0 (StataCorp).

### Ethical statement

This study involved human participants and was approved by the North West—Greater Manchester West Research Ethics Committee (REC reference: 18/NW/0613). Participants gave informed consent to participate in the study before taking part.

## RESULTS

**FIGURE 1:**
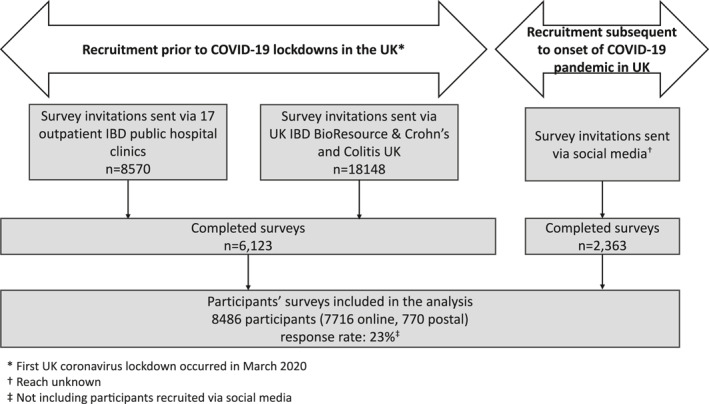
Recruitment of IBD‐BOOST Survey participants. IBD, inflammatory bowel disease.

In the IBD‐BOOST survey, over 26,000 people with IBD across the UK were approached; 8486 of them (7716 online, 770 postal) completed the IBD‐BOOST survey (response rate of 23%—Figure 1). Missing data were generally low (<5%), except for socioeconomic deprivation (11%), bowel incontinence (10%), and disease activity scores (11%–15%). Key demographic and clinical characteristics are presented in Table 1 (with further details in Tables S3 and S4). Most participants (91%) were white Caucasians and 49% of participants had CD. The participants' mean age was 50 years and their mean duration of IBD was 14 years. The EQ‐5D‐5L questionnaire was completed by 8153 participants, with 8121 (99.6%) of them responding to all five domains. Many participants reported problems across QoL domains (62% with pain/discomfort, 52% with anxiety/depression, 45% with usual activities, 33% with mobility, and 17% with self‐care). The mean QoL utility across the study participants was 0.76 (SD 0.23). As expected, participants with pain reported the highest percentage of problems in the pain/discomfort domain (97%) compared with those with fatigue (87%) and bowel incontinence (73%). This was also the case for problems with mobility (63% for participants with pain compared to 64% and 40% for participants with fatigue and bowel incontinence, respectively). Participants with fatigue reported the highest percentage of problems with self‐care (40%). The proportion of participants who reported problems with usual activities and anxiety/depression was similar among participants with fatigue or pain (79% and 76%, respectively) (Figure S1). For each IBD‐related symptom, the highest percentage of reported problems for any EQ‐5D domain was for pain/discomfort.

**TABLE 1:** Selected demographic and clinical characteristics of the 8486 IBD‐Boost survey participants.

Characteristic	Total (*n* = 8486)	
	*N*	(%) Or mean (SD)
IBD type		
Crohn's disease	4168	(49.1%)
Other IBD (including missing^a^)	4318	(50.1%)
Gender		
Male	3285	(38.7%)
Female	4888	(57.6%)
Missing	313	(3.7%)
Age (years)	49.8	(15.4)
Missing	304	(3.6%)
Ethnicity		
White	7751	(91.3%)
Mixed	118	(1.4%)
Asian	209	(2.5%)
Black	35	(0.4%)
Any other	71	(0.8%)
Missing	302	(3.6%)
Educational level		
No formal education	219	(2.6%)
School (GCSE or AS/A‐levels)	2277	(26.8%)
Further and higher education	5661	(66.7%)
Missing	329	(3.9%)
Employment status		
Employed	5071	(59.8%)
Unemployed due to illness	499	(5.9%)
Unemployed	218	(2.6%)
Student or homemaker	467	(5.5%)
Retired	1921	(22.6%)
Missing	310	(3.7%)
Relationship status		
Living alone	2378	(28.0%)
Living with someone	5779	(68.0%)
Missing	329	(3.9%)
IMD quintile		
1 (most deprived)	1124	(13.2%)
2	1357	(16.0%)
3	1611	(19.0%)
4	1709	(20.1%)
5 (least deprived)	1756	(20.7%)
Missing	929	(10.9%)
Pain (PROMIS score ≥60)	1769	(20.9%)
Missing	262	(3.1%)
Fatigue (PROMIS score ≥60)	2036	(24.0%)
Missing	42	(5.0%)
Bowel incontinence (PROMIS score ≥5)	4562	(53.8%)
Missing	839	(9.9%)
GAD‐7 anxiety level		
No anxiety (GAD‐7 score 0–4)	4353	(51.3%)
Mild anxiety (GAD‐7 score 5–9)	2097	(24.7%)
Moderate to severe anxiety (GAD‐7 score 10–21)	1733	(20.4%)
Missing	303	(3.6%)
PHQ‐9 depression level		
No depression (PHQ‐9 score 0–4)	3741	(44.1%)
Mild to moderate depression (PHQ‐9 score 5–9)	2132	(25.1%)
Moderately severe depression (PHQ‐9 score 10–27)	2306	(27.2%)
Missing	307	(3.6%)
IBD activity score (PRO‐2) for Crohn's disease	9.6	(10.4)
Missing	615	(14.8%)
IBD activity score (PRO‐2) for ulcerative colitis	1.2	(1.5)
Missing	456	(10.6%)
IBD control tertile		
1 (best control)	1903	(22.4%)
2	3291	(38.8%)
3 (worst control)	2862	(33.7%)
Missing	430	(5.0%)
EQ‐5D‐5L utility	0.76	(0.23)
Missing	385	(4.5%)

Abbreviations: BMI, body mass index; EQ‐5D‐5L, 5‐level EQ‐5D version; GAD‐7, Generalised Anxiety Disorder Assessment; GCSE, General Certificate of Secondary Education; IBD, inflammatory bowel disease; IMD, indices of multiple deprivation; PHQ‐9, Patient Health Questionnaire‐9; PRO‐2, Patient‐Reported Outcome; PROMIS, Patient‐Reported Outcomes Measurement Information System; SD, standard deviation.

^a^ Sixty‐six participants did not report their IBD diagnosis. Mean (SD) presented across participants with non‐missing data. IBD control tertiles (1: 15–16 [best control], 2: 9–14 and 3: 0–8 [worst control]).

**FIGURE 2:**
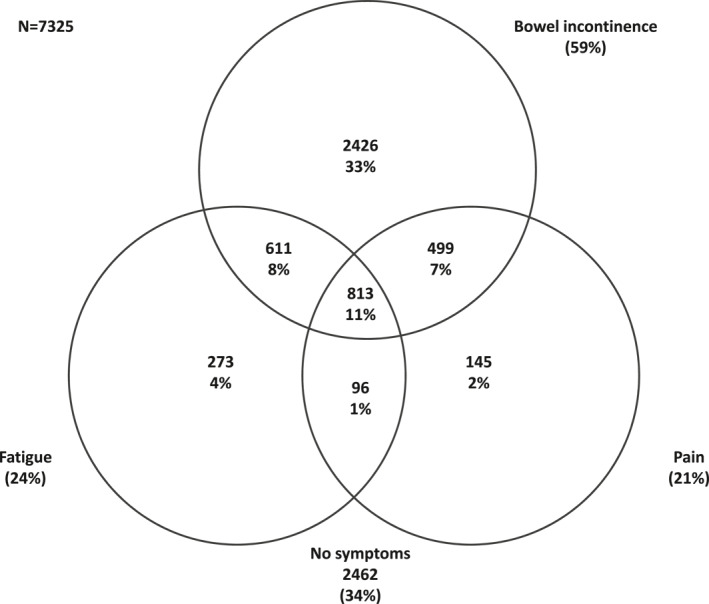
Co‐occurrence of pain, fatigue and bowel incontinence among IBD‐BOOST Survey participants with complete data. Participants with any missing data about fatigue, pain, and bowel incontinence symptoms (*n* = 1162) excluded. Number (%) presented. IBD, inflammatory bowel disease.

**FIGURE 3:**
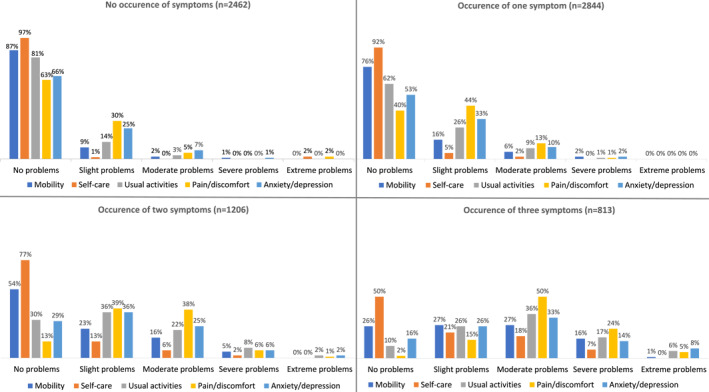
Distribution of responses to EQ‐5D‐5L questionnaire by (co‐)occurrence of pain, fatigue and bowel incontinence symptoms. Participants with any missing data about pain, fatigue and bowel incontinence symptoms (*n* = 1162) excluded.

Among participants with available symptom data, 59% experienced bowel incontinence, 24% fatigue, and 21% pain in the past week. While 33% of participants experienced bowel incontinence only, 4% fatigue only, 2% pain only, a substantial number of participants experienced multiple symptoms—8% experienced fatigue and incontinence, 7% pain and incontinence, 1% fatigue and pain, and 11% of participants reported all three symptoms in the past week (Figure 2). Participants experiencing pain, fatigue or bowel incontinence reported issues across QoL domains, with participants who experienced co‐occurring symptoms reporting lower QoL. Specifically, of the 813 participants (11%) who experienced all three symptoms of pain, fatigue and bowel incontinence, severe or extreme problems were reported for the pain/discomfort domain by 29%, for usual activity by 23%, for anxiety/depression by 22%, for mobility by 17%, and for self‐care by 7% (Figure 3).

### QoL of people living with IBD

In the initial QoL model of people living with IBD, the QoL of the reference individual—a 50‐year old man with CD who is a non‐smoker, has a normal weight, lives alone in an area with socio‐economic deprivation in quintile 3, educated to General Certificate of Secondary Education (GCSE) or Advanced Subsidiary (AS) /A (Advanced)‐levels, is employed, has no history of IBD surgery and possesses no physical and mental health comorbidities—was estimated at 0.862 (standard error [SE] 0.011) (Table S5). Being female, unemployed, a smoker, being outside the normal BMI weight range, living alone, using biological treatment and having physical or mental comorbidities were all independently associated with lower QoL. In particular, being unemployed due to illness was associated with a large QoL decrement (−0.321 [SE 0.010]) relative to being employed and living with someone was associated with higher QoL (0.026 [0.005]) relative to living alone. All else equal, QoL was similar between people living with CD and other IBD (Table S5).

### Decrements in QoL associated with symptoms of pain, fatigue and bowel incontinence (stage 1)

Adding dichotomised pain, fatigue and bowel incontinence symptoms in the model were all associated with significant QoL decrements. Pain was associated with the largest QoL utility decrement (−0.159 [SE 0.005]), followed by fatigue (−0.140 [SE 0.005]) and bowel incontinence (−0.048 [SE 0.004]) (Table 2—Stage 1). Clear associations between the level of symptom severity and QoL utility were also observed. Compared with a PROMIS symptom score <50 for pain and fatigue, a PROMIS score ≥60 was associated with a QoL decrement of −0.184 (SE 0.006) for pain and −0.161 (SE 0.006) for fatigue (both *p*_trend_ < 0.001). A PROMIS bowel incontinence score ≥10 was associated with a QoL decrement of −0.063 (SE 0.006) compared with a PROMIS bowel incontinence score <5 (*p*_trend_ < 0.001) (Table S6).

**TABLE 2:** Quality of life (EQ‐5D utility) associated with patient characteristics and key symptoms of inflammatory bowel disease (*N* = 8486 participants).

Characteristic	Stage 1	Stage 2	Stage 3
	Mean (SE)	Mean (SE)	Mean (SE)
QoL of reference individual^a^	0.928 (0.009)*	0.927 (0.009)*	0.960 (0.009)*
Female sex	−0.007 (0.004)	−0.008 (0.004)*	−0.004 (0.004)
Age at time of EQ‐5D questionnaire (per 10 years, centred at 50)	0.006 (0.002)*	0.006 (0.002)*	−0.002 (0.002)
Time since diagnosis (ref: 0–3 years)			
4 years and over	0.004 (0.005)	0.004 (0.005)	−0.003 (0.005)
Pregnant	0.012 (0.023)	0.013 (0.023)	−0.002 (0.022)
Smoking status (ref: never smoker)			
Ex‐smoker	−0.015 (0.004)*	−0.016 (0.004)*	−0.013 (0.004)*
Current smoker	−0.028 (0.007)*	−0.028 (0.007)*	−0.018 (0.007)*
BMI (ref: normal weight)			
Underweight	−0.036 (0.012)*	−0.035 (0.012)*	−0.029 (0.011)*
Overweight	−0.020 (0.004)*	−0.020 (0.004)*	−0.016 (0.004)*
IMD quintile (ref: 3)			
1	−0.011 (0.007)	−0.011 (0.007)	−0.008 (0.006)
2	0.003 (0.006)	0.003 (0.006)	0.001 (0.006)
4	−0.002 (0.006)	−0.002 (0.006)	−0.001 (0.006)
5	0.001 (0.006)	0.000 (0.006)	0.002 (0.005)
Education level (ref: GCSE or AS/A‐levels)			
No education	−0.016 (0.012)	−0.016 (0.012)	−0.009 (0.011)
Further and higher education	0.003 (0.004)	0.003 (0.004)	0.002 (0.004)
Employment status (ref: employed)			
Unemployed due to illness	−0.216 (0.008)*	−0.215 (0.008)*	−0.201 (0.008)*
Unemployed	−0.066 (0.012)*	−0.065 (0.012)*	−0.055 (0.011)*
Student or homemaker	−0.015 (0.008)	−0.015 (0.008)	−0.016 (0.007)*
Retired	−0.033 (0.006)*	−0.033 (0.006)*	−0.038 (0.005)*
Living circumstances (ref: living alone)			
Living with someone	0.018 (0.004)*	0.018 (0.004)*	0.012 (0.004)*
IBD type (ref: Crohn's disease)			
Other IBD	−0.001 (0.004)	−0.001 (0.004)	−0.001 (0.004)
IBD operation	−0.003 (0.005)	−0.002 (0.005)	−0.005 (0.004)
Use of biologic medication	−0.006 (0.004)	−0.006 (0.004)	−0.003 (0.004)
Physical comorbidity	−0.031 (0.004)*	−0.031 (0.004)	−0.028 (0.004)*
Mental comorbidity	−0.096 (0.005)*	−0.096 (0.005)	−0.055 (0.004)*
PROMIS pain	−0.159 (0.005)*	−0.145 (0.007)*	−0.106 (0.006)*
PROMIS fatigue	−0.140 (0.005)*	−0.130 (0.006)*	−0.064 (0.006)*
PROMIS bowel incontinence	−0.048 (0.004)*	−0.049 (0.004)*	−0.028 (0.004)*
PROMIS pain AND PROMIS fatigue		−0.032 (0.010)*	−0.049 (0.009)*
GAD‐7 scale for anxiety (ref: no anxiety)**			
Mild anxiety			−0.038 (0.005)*
Moderate to severe anxiety			−0.102 (0.006)*
PHQ‐9 scale for depression (ref: no depression)**			
Mild to moderate depression			−0.050 (0.005)*
Moderately severe depression			−0.110 (0.007)*

*Note*: PROMIS pain (≥60); PROMIS fatigue (≥60); PROMIS bowel incontinence (≥5); *p‐value <0.05; **p_trend_ < 0.05. Interactions between fatigue and bowel incontinence and pain and bowel incontinence (Stage 2) excluded as not statistically significant.

Abbreviations: BMI, body mass index; EQ‐5D‐5L, 5‐level EQ‐5D version; GAD‐7, Generalised Anxiety Disorder Assessment; IBD, inflammatory bowel disease; IMD, indices of multiple deprivation; PHQ‐9, Patient Health Questionnaire‐9; PROMIS, Patient‐Reported Outcomes Measurement Information System; SD, standard deviation.

^a^ Fifty‐year‐old man with Crohn's disease, diagnosed in the last 3 years, not pregnant, without operation and not receiving biological medication, never smoker, normal weight, living alone and in an area of average socioeconomic deprivation, educated at GCSE or AS/A levels, employed, without physical or mental health comorbidities, and, in models Stage 2 and 3, without respective symptoms.

### Decrements in QoL accounting for interactions between fatigue, pain, and bowel incontinence (stage 2)

At stage 2, the interactions between pain, fatigue and bowel incontinence were added to the QoL model (Table 2—Stage 2). The three‐way interaction between the symptoms and the interactions between fatigue and bowel incontinence and pain and bowel incontinence were not statistically significant and thus excluded. Strong interaction was observed only between pain and fatigue (−0.032 [SE 0.010]) indicating even larger reductions in QoL among patients experiencing these co‐occurring symptoms.

### Further contribution of anxiety and depression to reduced QoL (stage 3)

At stage 3, adjusting for anxiety and depression only marginally weakened the associations of other patient characteristics and symptoms with QoL. Adding anxiety and depression into the model indicated that they were both associated with lower QoL (Table 2—Stage 3) with graded associations across levels of symptom severity (both *p*_trend_ < 0.05). Severe levels of depression (−0.110 [SE 0.007]) and anxiety (−0.102 [SE 0.006]) were associated with the largest reductions in QoL utility. In a further analysis exploring interactions between pain, fatigue, bowel incontinence, anxiety, and depression, a significant interaction between anxiety and fatigue was noted (Table S7).

### Sensitivity analyses

Higher levels of IBD activity and lower levels of IBD control were both associated with significantly lower QoL and their inclusion in QoL models marginally weakened the associations between symptoms and QoL (Table S8).

Similar decrements in QoL associated with pain, fatigue, bowel incontinence, anxiety and depression were observed in separate analyses among participants with CD and other types of IBD (Table S9), and separately among women and men (Table S10).

## DISCUSSION

In this study, we estimated decrements in QoL associated with IBD‐related symptoms of pain, fatigue, and bowel incontinence, and the further contributions of anxiety and depression. We found that pain and fatigue were associated with larger QoL decrements compared with bowel incontinence. We also identified clear graded associations between symptom severity and QoL, suggesting that people with IBD who experience more severe symptoms have lower QoL. We noted significant negative interactions between pain and fatigue, suggesting that the co‐occurrence of these symptoms was associated with even larger decrements in QoL. Anxiety and depression were shown to further impact the QoL of people living with IBD.

To our knowledge, there are no studies assessing decrements in QoL associated with pain, fatigue or bowel incontinence in IBD using the EuroQoL EQ‐5D generic health‐related QoL instrument; therefore, no direct comparisons can be made with our study. Our study is the first to provide estimates of QoL associated with the common symptoms of pain, fatigue and bowel incontinence, and interactions between them in patients with IBD. Although the associations of pain and fatigue with QoL are somewhat larger than the association between bowel incontinence and QoL, given the high frequency of bowel incontinence reported in this study, the overall impact of this symptom on QoL is substantial. Our statistical model also highlighted the relationship between anxiety and depression and QoL in people with IBD.

In line with other studies,^25^ we found no significant difference between the QoL of participants diagnosed with CD compared with participants with other types of IBD. Similar to other studies,^26^ we also reported that being unemployed and living alone were associated with lower QoL. Other sociodemographic factors identified in our study associated with lower QoL included having a BMI outside the healthy weight range and being a smoker.

Two previous studies using the SF‐36^27^ and an IBD‐specific QoL instrument (IBDQ‐9),^28^ respectively, reported that fatigue is associated with impaired QoL. Similarly, a study using the short inflammatory bowel disease questionnaire,^29^ reported a significant correlation between pain and reduced QoL in IBD. An association between bowel incontinence and lower QoL, measured using IBD‐Q, has also been reported.^30^ The interaction between pain and fatigue in IBD has also been previously highlighted.^31^ Although individual associations between symptoms of pain, fatigue and bowel incontinence with QoL have been noted, our study is the first to quantify the joint impact of these symptoms and their co‐occurrence on QoL using generic HRQoL measure.

A much larger proportion of IBD patients report anxiety and/or depression compared to the general population.^32^ In our study, 49% of respondents reported suffering from anxiety and 56% from depression, while for the general population the levels are much lower at 6% and 3%, respectively.^32^ The current study found that severe levels of depression and anxiety were associated with substantial QoL decrements.

Our study aligns with previous reports in IBD^33^, ^34^ indicating that IBD activity and poor IBD control are significant contributors to lower QoL. IBD activity and control are also associated with the symptoms studied and therefore, we initially excluded them to allow more comprehensive assessment of the associations between symptoms with QoL. However, although the associations between symptoms and QoL were somewhat reduced following adjustment for disease activity and control, they remained substantial, indicating the need for improved symptom management in addition to managing disease activity and control.

We also compared the study estimates of symptoms' impact on QoL in IBD with other chronic conditions that used EQ‐5D‐5L to measure QoL utility. Specifically, a study examining the associations between symptoms and QoL in people with multiple sclerosis indicated that pain, fatigue, anxiety, and depression all had substantial negative impacts on QoL.^35^ Comparing the associations of anxiety and depression with QoL with other chronic conditions, a study investigating the QoL of adult patients with rheumatoid arthritis,^36^ found a negative correlation between anxiety and depression and HRQoL. These results concur with the findings from our study, showing that pain and fatigue are common symptoms and significant predictors of HRQoL in chronic diseases, and that anxiety and depression emerge as important contributors to HRQoL in these populations.

Several limitations of the present study need to be acknowledged. First, in view of the cross‐sectional study design, we cannot be confident that the estimated associations between symptoms and QoL are causal. Further prospective longitudinal studies are needed to strengthen the evidence. Second, the present study did not include a control group and therefore we are unable to investigate in detail whether associations between symptoms such as depression and anxiety and QoL in IBD are similar to those reported in other populations. Third, the study was carried out during the COVID‐19 pandemic in the UK and required a change in recruitment method from hospital outpatient databases to self‐selection via social media. Therefore, there is some risk of participants with a non‐verified IBD diagnosis being included in the study. Fourth, the study did not aim to recruit a representative sample of the UK IBD patient population. The study sample, for example, is likely to include a higher proportion of hospital‐managed IBD patients (as IBD Bioresource recruited from hospitals). However, the IBD‐BOOST's large and well‐characterised survey population allowed adjustments for a wide range of individual patient characteristics and the study results are expected to generalise to a wider IBD patient population. Lastly, in a large‐scale online survey such as this, it was impossible to have an objective marker of IBD activity such as faecal calprotectin, and we recognise that self‐reported disease activity has a low correlation with endoscopic activity, making our PRO definition of disease activity liable to confounding by symptoms such as those attributable to irritable bowel syndrome.

In conclusion, we report, for the first time, detailed estimates of decrements in QoL associated with the symptoms of pain, fatigue and bowel incontinence in IBD. We also highlight the associations of anxiety and depression with QoL in the IBD population. These findings add to the existing literature^37^ for the need for change in IBD management practice, with increased focus on directly managing these symptoms given that their negative impacts on QoL goes beyond the impact mediated through IBD disease activity and control. New interventions to better manage these symptoms in people with IBD are likely to improve their QoL.

## AUTHOR CONTRIBUTIONS

Borislava Mihaylova, Chris Roukas and Christine Norton conceived and designed the study. Chris Roukas, Laura Miller, Thomas Hamborg, Imogen Stagg, Alisa Hart, Christine Norton and Borislava Mihaylova contributed to the acquisition of data, and Chris Roukas, Fionn Cléirigh Büttner, Thomas Hamborg and Borislava Mihaylova analysed the data. All authors contributed to the interpretation of the data, the development of the manuscript, and critically reviewed/revised the manuscript for important intellectual content. All authors approved the final version of the manuscript before submission.

## CONFLICT OF INTEREST STATEMENT

CN declares the following conflicts of interest: Speaker fees from Janssen, WebMD, Medscape, Merck Pharmaceutical, Tillotts Pharma UK. Pfizer advisory board. AH reports consulting fees from AbbVie, BMS, Celltrion, Dr Falk Pharma, Galapagos, Lilly, Janssen, Pfizer and Takeda and has received speaker fees and sponsorship for academic meetings from Abbvie, BMS, Celltrion, Galapagos, Lilly, Jannsen, Pfizer and Takeda. JOL served as a consultant and an advisory board participant for AbbVie, Bristol Myers Squibb, Celgene, Celltrion, Eli Lilly, Engytix, Ferring Pharmaceuticals, Galapagos, Gilead, GSK, Janssen, MSD, Napp, Pfizer, Shire, Takeda, and Vifor Pharma; has received speaker fees and sponsorship for academic meetings from AbbVie, BMS, Celltrion, Ferring Pharmaceuticals, Janssen, MSD, Napp, Norgine, Pfizer, Shire, Tillotts Pharma, and Takeda; and has received investigator‐led research grants from AbbVie, Gilead, Pfizer, Shire, and Takeda. Other Authors declare no conflicts of interest related to this work.

## Supporting information

Supporting Information S1

## Data Availability

The data that support the findings of this study are available from Prof. Christine Norton on reasonable request. The data are not publicly available due to privacy or ethical restrictions.
