# Supplementary material for: Impact of pain, fatigue and bowel incontinence on the quality of life of people living with inflammatory bowel disease: A UK cross‐sectional survey
Source: United European Gastroenterol J. 2024 Oct 19;13(3):364–75. doi: 10.1002/ueg2.12668 (PMC7616848; doi:10.1002/ueg2.12668)
Supplement: Supplementary file 1 — Supporting Information S1 [file UEG2-13-364-s001.docx]

**Impact of pain, fatigue and bowel incontinence on the quality of life of people living with inflammatory bowel disease: a UK cross-sectional survey**

**Supplementary material**

[Supplementary Figure S1: Distribution of EQ-5D-5L domains by IBD-related reported symptom 2](#_Toc178365018)

[Supplementary Table S1: Definitions of variables included for selection in the final statistical model 4](#_Toc178365019)

[Supplementary Table S2: Model specifications for impact of IBD symptoms on HRQoL 6](#_Toc178365020)

[Supplementary Table S3: Socio-demographic characteristics of the 8486 IBD-BOOST survey participants 9](#_Toc178365021)

[Supplementary Table S4: Clinical characteristics of 8486 participants included in the IBD-BOOST survey by IBD diagnosis 11](#_Toc178365022)

[Supplementary Table S5: Quality of life (EQ-5D utility) associated with IBD patients’ socio-demographic and clinical characteristics (Initial QoL model) 13](#_Toc178365023)

[Supplementary Table S6: Quality of life (EQ-5D utility) associated with IBD patients’ socio-demographic, clinical characteristics and severity of symptoms of pain, fatigue and bowel incontinence (N=8486 participants) 14](#_Toc178365024)

[Supplementary Table S7: Quality of life (EQ-5D utility) associated with IBD patients’ socio-demographic, clinical characteristics, symptoms of pain, fatigue and bowel incontinence and interactions with anxiety and depression (N=8486 participants) 16](#_Toc178365025)

[Supplementary Table S8: Quality of life (EQ-5D utility) associated with IBD patients’ socio-demographic, clinical characteristics, symptoms of pain, fatigue and bowel incontinence, anxiety and depressions, including adjustments for IBD activity and control (N=8486) 18](#_Toc178365026)

[Supplementary Table S9: Quality of life (EQ-5D utility) associated with socio-demographic, clinical characteristics, symptoms of pain, fatigue and bowel incontinence and with anxiety and depression, by IBD type 21](#_Toc178365027)

[Supplementary Table S10: Quality of life (EQ-5D utility) associated with socio-demographic, clinical characteristics, symptoms of pain, fatigue and bowel incontinence and with anxiety and depression, by gender 24](#_Toc178365028)

## Supplementary Figure S1: Distribution of EQ-5D-5L domains by IBD-related reported symptom

1. EQ-5D domains among IBD participants with pain


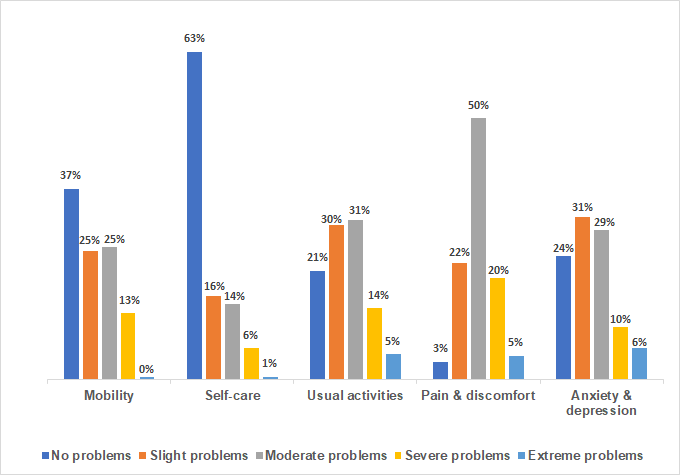


1. EQ-5D domains among IBD participants with fatigue


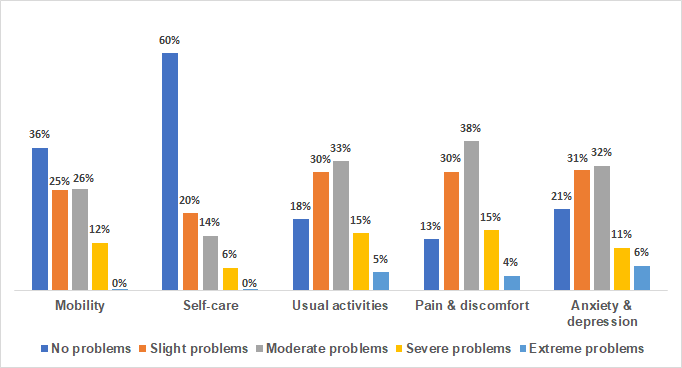


1. EQ-5D domains among IBD participants with bowel incontinence


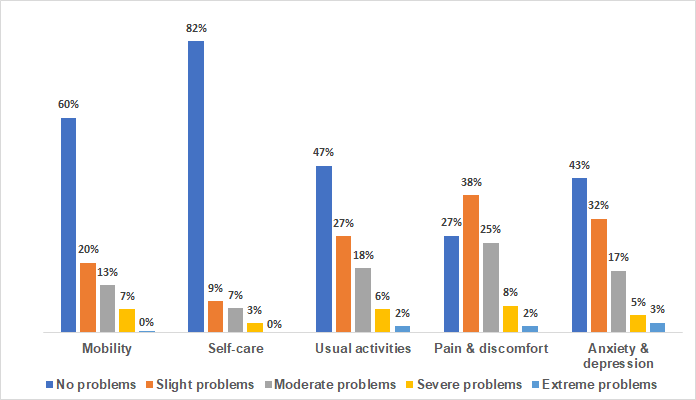


## Supplementary Table S1: Definitions of variables included for selection in the final statistical model

| Variables | Categories (if categorical) | Note |
| --- | --- | --- |
| IBD type | Crohn’s disease or Crohn’s colitis  Other type of IBD |  |
| Age | 18-25, 26-35, 36-45, 46-55, 56-65, 66 and over |  |
| Gender | Male, Female |  |
| Smoking status | Never smoked, Ex-smoker, Current smoker |  |
| IMD quintile | 1 (most deprived), 2, 3, 4, 5 (least deprived) | Measure of socioeconomic status calculated using self-reported postcode |
| BMI (kg/m^2^) | <25, >=25-<30, >=30-<35, >=35 | Based on self-reported height and weight |
| IBD surgery in the past | Yes/No |  |
| IBD biologic medication | Yes/No |  |
| IBD activity score or Stoma/pouch | 1 (low activity), 2, 3 (high activity), 4 (stoma/pouch) | Tertiles in study population |
| IBD control score | 1 (best control), 2,3 (worst control) | Tertiles in study population |
| Physical health comorbidity | Yes/No |  |
| Mental health comorbidity | Yes/No |  |
| Time since IBD diagnosis (in years) | 0-3, >4 |  |
| Currently pregnant | Yes/No |  |
| Education level | No formal education, School (GCSE or AS/A-levels), Further and higher education |  |
| Employment status | Employed, Unemployed due to illness, Unemployed, Student or homemaker, Retired |  |
| Relationship status | Living alone, Living with someone |  |
| Pain | 0-59 (no pain), 60 and over (pain) | PROMIS pain score (dichotomised) |
| Fatigue | 0-59 (no fatigue), 60 and over (fatigue) | PROMIS fatigue score (dichotomised) |
| Bowel Incontinence | 0-4 (no bowel incontinence), 5-20 (bowel incontinence) | PROMIS Gastrointestinal Bowel Incontinence score (dichotomised) |
| Anxiety | 0-4 (no anxiety), 5-9 (mild anxiety), 10-21 (moderate to severe anxiety) | GAD-7 scale for anxiety |
| Depression | 0-4 (no depression), 5-9 (mild to moderate depression), 10-27(moderately severe depression) | PHQ-9 scale for depression |

Abbreviations: IBD: Inflammatory bowel disease; IMD: Indices of Multiple Deprivation; BMI: Body Mass Index; PROMIS: Patient-Reported Outcomes Measurement Information System; GAD-7: Generalised Anxiety Disorder Assessment; PHQ-9: Patient Health Questionnaire.

## Supplementary Table S2: Model specifications for impact of IBD symptoms on HRQoL

| **Outcome** | **Model** | **Key exposures** | **Further predictors** | **Confounders:** |
| --- | --- | --- | --- | --- |
| EQ-5D-5L utility | Stage 1 | - PROMIS pain intensity score^1^ - PROMIS fatigue score^1^ - PROMIS bowel incontinence score^1^ | Core (retained in all models)   - IBD type (Crohn’s vs other IBD) - Had IBD surgery in past (yes, no) - IBD biologic medication (yes/no)   Retain only if statistically significant (p<0.05):   - Time since diagnosis (0-3 years vs 4+ years) | Core (retained in all models)   - Age (years) ^1^ - Gender - Smoking status (never, ex, current) - BMI (underweight, normal, overweight) - IMD quintile (1: least deprived to 5: most deprived) - Physical health comorbidity (yes/no) - Mental health comorbidity (yes/no)   Enter one at a time in this order and retain only if statistically significant (p<0.05):   - Currently pregnant (yes/no) - Education (no formal education, school (GCSE or AS/A-levels), “further & higher education”) - Employment (paid employment, unemployed due to illness/disease, all other) - Relationship status (in relationship vs not) |
| EQ-5D-5L utility | Stage 2 | Continuous or categorical (as indicated in Model 1):   - PROMIS pain intensity - PROMIS fatigue - PROMIS bowel incontinence   Add all interaction terms between symptoms where more than 20% overlap in symptoms present.  Retain interactions only if statistically significant (p<0.05). | - IBD Type (Crohn’s vs other IBD) - Had IBD surgery in past (yes, no) - IBD biologic medication (yes/no) - (if statistically significant) Time since diagnosis (0-3 years vs 4+ years) | Core (retained in all models)   - Age (years)* - Gender - Smoking status (never, ex, current) - BMI (underweight, normal, overweight) - IMD quintile (1: least deprived to 5: most deprived) - Physical health comorbidity (yes/no) - Mental health comorbidity (yes/no)   Those retained from model stage 1 only from:   - Currently pregnant (yes/no) - Education (no formal education, school (GCSE or AS/A-levels), “further & higher education”) - Employment (paid employment, unemployed due to illness/disease, all other) - Relationship status (in relationship vs not) |
| EQ-5D-5L utility | Stage 3 | Continuous or categorical (as indicated in Model 1):   - PROMIS pain intensity - PROMIS fatigue - PROMIS bowel incontinence   Retained interactions between pain, fatigue, and bowel incontinence.  Further symptoms:   - GAD-7 score for anxiety (no anxiety; mild; moderate to severe anxiety) - PHQ-9 score for depression (no depression; mild to moderate; moderately severe depression)   Add all interaction terms between the three key symptoms and anxiety and depression where more than 20% overlap symptoms present. Retain interactions only if statistically significant (p<0.05). | - IBD Type (Crohn’s vs other IBD) - Had IBD surgery in past (yes, no) - IBD biologic medication (yes/no) - (if statistically significant) Time since diagnosis (0-3 years vs 4+ years) | Core (retained in all models)   - Age (years)* - Gender - Smoking status (never, ex, current) - BMI (underweight, normal, overweight) - IMD quintile (1: least deprived to 5: most deprived) - Physical health comorbidity (yes/no) - Mental health comorbidity (yes/no)   Those retained from model stage 1 only from:   - Currently pregnant (yes/no) - Education (no formal education, school (GCSE or AS/A-levels), “further & higher education”) - Employment (paid employment, unemployed due to illness/disease, all other) - Relationship status (in relationship vs not) |

^1^Nonlinearility in continuous variables will be checked in final models and, if necessary, addressed using categorisation (symptom scores) or spline functions (age)

IBD: Inflammatory Bowel Disease; IMD: Indices of Multiple Deprivation; BMI: Body Mass Index; EQ-5D-5L: 5-level EQ-5D version; PROMIS: Patient-Reported Outcomes Measurement Information System; GAD-7: Generalised Anxiety Disorder Assessment; PHQ-9: Patient Health Questionnaire.

## Supplementary Table S3: Socio-demographic characteristics of the 8486 IBD-BOOST survey participants

| **Characteristic** | **Type of IBD** | | | | | |
| --- | --- | --- | --- | --- | --- | --- |
|  | **Crohn's Disease**  **(n=4168)** | | **Other IBD**  **(n=4318)** | | **Total**  **(n=8486)** | |
| **Gender** |  |  |  |  |  |  |
| Male | 1479 | (35.5%) | 1806 | (41.8%) | 3285 | (38.7%) |
| Female | 2550 | (61.2%) | 2338 | (54.1%) | 4888 | (57.6%) |
| Missing | 139 | (3.3%) | 174 | (4.0%) | 313 | (3.7%) |
| **Participant age (in years) mean (SD)** | 48.1 | (15.1) | 51.4 | (15.6) | 49.8 | (15.4) |
| **Age group** |  |  |  |  |  |  |
| 18-25 | 294 | (7.1%) | 201 | (4.7%) | 495 | (5.8%) |
| 26-35 | 688 | (16.5%) | 606 | (14.0%) | 1294 | (15.2%) |
| 36-45 | 759 | (18.2%) | 751 | (17.4%) | 1510 | (17.8%) |
| 46-55 | 917 | (22.0%) | 758 | (17.6%) | 1675 | (19.7%) |
| 56-65 | 803 | (19.3%) | 924 | (21.4%) | 1727 | (20.4%) |
| 66 over | 570 | (13.7%) | 911 | (21.1%) | 1481 | (17.5%) |
| Missing | 137 | (3.3%) | 167 | (3.9%) | 304 | (3.6%) |
| **Ethnicity** |  |  |  |  |  |  |
| White | 3840 | (92.1%) | 3911 | (90.6%) | 7751 | (91.3%) |
| Mixed | 60 | (1.4%) | 58 | (1.3%) | 118 | (1.4%) |
| Asian | 78 | (1.9%) | 131 | (3.0%) | 209 | (2.5%) |
| Black | 22 | (0.5%) | 13 | (0.3%) | 35 | (0.4%) |
| Any other | 31 | (0.7%) | 40 | (0.9%) | 71 | (0.8%) |
| Missing | 137 | (3.3%) | 165 | (3.8%) | 302 | (3.6%) |
| **Educational level** |  |  |  |  |  |  |
| No formal education | 98 | (2.4%) | 121 | (2.8%) | 219 | (2.6%) |
| School (GCSE or AS/A-levels) | 1166 | (28.0%) | 1111 | (25.7%) | 2277 | (26.8%) |
| Further and Higher education | 2753 | (66.1%) | 2908 | (67.3%) | 5661 | (66.7%) |
| Missing | 151 | (3.6%) | 178 | (4.1%) | 329 | (3.9%) |
| **Employment status** |  |  |  |  |  |  |
| Employed | 2540 | (60.9%) | 2531 | (58.6%) | 5071 | (59.8%) |
| Unemployed due to illness | 330 | (7.9%) | 169 | (3.9%) | 499 | (5.9%) |
| Unemployed | 119 | (2.9%) | 99 | (2.3%) | 218 | (2.6%) |
| Student or Homemaker | 258 | (6.2%) | 209 | (4.8%) | 467 | (5.5%) |
| Retired | 778 | (18.7%) | 1143 | (26.5%) | 1921 | (22.6%) |
| Missing | 143 | (3.4%) | 167 | (3.9%) | 310 | (3.7%) |
| **Relationship status** |  |  |  |  |  |  |
| Living alone | 1260 | (30.2%) | 1118 | (25.9%) | 2378 | (28.0%) |
| Living with someone | 2756 | (66.1%) | 3023 | (70.0%) | 5779 | (68.1%) |
| Missing | 152 | (3.6%) | 177 | (4.1%) | 329 | (3.9%) |
| **IMD quintile** |  |  |  |  |  |  |
| 1 (most deprived) | 550 | (13.2%) | 574 | (13.3%) | 1124 | (13.2%) |
| 2 | 662 | (15.9%) | 695 | (16.1%) | 1357 | (16.0%) |
| 3 | 795 | (19.1%) | 816 | (18.9%) | 1611 | (19.0%) |
| 4 | 833 | (20.0%) | 876 | (20.3%) | 1709 | (20.1%) |
| 5 (least deprived) | 859 | (20.6%) | 897 | (20.8%) | 1756 | (20.7%) |
| Missing | 469 | (11.3%) | 460 | (10.7%) | 929 | (10.9%) |
| **Smoking status** |  |  |  |  |  |  |
| Never smoked | 2072 | (49.7%) | 2093 | (48.5%) | 4165 | (49.1%) |
| Ex-smoker | 1577 | (37.8%) | 1887 | (43.7%) | 3464 | (40.8%) |
| Current smoker | 367 | (8.8%) | 162 | (3.8%) | 529 | (6.2%) |
| Missing | 152 | (3.6%) | 176 | (4.1%) | 328 | (3.9%) |
| **Alcohol intake** |  |  |  |  |  |  |
| Never | 1931 | (46.3%) | 1556 | (36.0%) | 3487 | (41.1%) |
| 1-14 units | 1817 | (43.6%) | 2179 | (50.5%) | 3996 | (47.1%) |
| 15 or more units | 268 | (6.4%) | 406 | (9.4%) | 674 | (7.9%) |
| Missing | 152 | (3.6%) | 177 | (4.1%) | 329 | (3.9%) |

IBD: Inflammatory bowel disease; IMD: Indices of Multiple Deprivation; SD: Standard Deviation; BMI: Body Mass Index; EQ-5D-5L: 5-level EQ-5D version; PROMIS: Patient-Reported Outcomes Measurement Information System; GAD-7: Generalised Anxiety Disorder Assessment; PHQ-9: Patient Health Questionnaire

## Supplementary Table S4: Clinical characteristics of 8486 participants included in the IBD-BOOST survey by IBD diagnosis

| **Characteristic** | **Type of IBD** | | | | | |
| --- | --- | --- | --- | --- | --- | --- |
|  | **Crohn's Disease**  **(n=4168)** | | **Other IBD**  **(n=4318)** | | **Total**  **(n=8486)** | |
| **BMI (kg/m^2^) – mean (SD)** | 26.8 | (6.0) | 26.9 | (5.8) | 26.8 | (5.9) |
| **BMI ranges** |  |  |  |  |  |  |
| Healthy Weight, n(%) | 1625 | (39.0%) | 1590 | (36.8%) | 3215 | (37.9%) |
| Underweight, n(%) | 121 | (2.9%) | 74 | (1.7%) | 195 | (2.3%) |
| Overweight, n(%) | 2151 | (51.6%) | 2321 | (53.8%) | 4472 | (52.7%) |
| Missing, n(%) | 271 | (6.5%) | 333 | (7.7%) | 604 | (7.1%) |
| **Operation for IBD in the past** |  |  |  |  |  |  |
| No, n(%) | 1930 | (46.3%) | 3805 | (88.1%) | 5735 | (67.6%) |
| Yes, n(%) | 2142 | (51.4%) | 398 | (9.2%) | 2540 | (29.9%) |
| Missing, n(%) | 96 | (2.3%) | 115 | (2.7%) | 211 | (2.5%) |
| **Having stoma** |  |  |  |  |  |  |
| No, n(%) | 3761 | (90.2%) | 4087 | (94.7%) | 7848 | (92.5%) |
| Yes, n(%) | 382 | (9.2%) | 167 | (3.9%) | 549 | (6.5%) |
| Missing, n(%) | 25 | (0.6%) | 64 | (1.5%) | 89 | (1.0%) |
| **Having pouch** |  |  |  |  |  |  |
| No, n(%) | 4061 | (97.4%) | 4114 | (95.3%) | 8175 | (96.3%) |
| Yes, n(%) | 73 | (1.8%) | 136 | (3.1%) | 209 | (2.5%) |
| Missing , n(%) | 34 | (0.8%) | 68 | (1.6%) | 102 | (1.2%) |
| **Had fistula surgery in the past** |  |  |  |  |  |  |
| 0 , n(%) | 3340 | (80.1%) | 3837 | (88.9%) | 7177 | (84.6%) |
| 1, n(%) | 808 | (19.4%) | 425 | (9.8%) | 1233 | (14.5%) |
| Missing, n(%) | 20 | (0.5%) | 56 | (1.3%) | 76 | (0.9%) |
| **Biologic medications** |  |  |  |  |  |  |
| No, n(%) | 1667 | (40.0%) | 2425 | (56.2%) | 4092 | (48.2%) |
| Yes, n(%) | 1925 | (46.2%) | 983 | (22.8%) | 2908 | (34.3%) |
| Missing, n(%) | 576 | (13.8%) | 910 | (21.1%) | 1486 | (17.5%) |
| **Having physical comorbidities** |  |  |  |  |  |  |
| No, n(%) | 2617 | (62.8%) | 2621 | (60.7%) | 5238 | (61.7%) |
| Yes, n(%) | 1551 | (37.2%) | 1697 | (39.3%) | 3248 | (38.3%) |
| **Having mental health comorbidities** |  |  |  |  |  |  |
| No, n(%) | 3085 | (74.0%) | 3444 | (79.8%) | 6529 | (76.9%) |
| Yes, n(%) | 1083 | (26.0%) | 874 | (20.2%) | 1957 | (23.1%) |
| **Pregnant** |  |  |  |  |  |  |
| No, n(%) | 3902 | (93.6%) | 3994 | (92.5%) | 7896 | (93.0%) |
| Yes, n(%) | 37 | (0.9%) | 26 | (0.6%) | 63 | (0.7%) |
| Missing, n(%) | 229 | (5.5%) | 298 | (6.9%) | 527 | (6.2%) |
| **PROMIS pain intensity T-score – mean (SD)** | 51.3 | (11.3) | 48.4 | (10.9) | 49.8 | (11.2) |
| **Pain levels** |  |  |  |  |  |  |
| 0-49, n(%) | 1785 | (42.8%) | 2240 | (51.9%) | 4025 | (47.4%) |
| 50-59, n(%) | 1252 | (30.0%) | 1178 | (27.3%) | 2430 | (28.6%) |
| 60 and over, n(%) | 1041 | (25.0%) | 728 | (16.7%) | 1769 | (20.9%) |
| Missing, n(%) | 90 | (2.2%) | 172 | (3.9%) | 262 | (3.1%) |
| **PROMIS fatigue T-score – mean (SD)** | 54.8 | (8.8) | 52.4 | (8.9) | 53.6 | (9.0) |
| **Fatigue levels** |  |  |  |  |  |  |
| 0-49 (mild), n(%) | 1144 | (27.5%) | 1618 | (37.5%) | 2762 | (32.6%) |
| 50-59 (moderate) , n(%) | 1653 | (39.7%) | 1609 | (37.3%) | 3262 | (38.4%) |
| 60 and over (severe) , n(%) | 1186 | (28.5%) | 850 | (19.7%) | 2036 | (24.0%) |
| Missing, n(%) | 185 | (4.4%) | 241 | (5.6%) | 426 | (5.0%) |
| **PROMIS bowel incontinence (raw) score – mean (SD)** | 6.8 | (3.5) | 6.4 | (3.4) | 6.6 | (3.4) |
| **Bowel incontinence levels** |  |  |  |  |  |  |
| 0-4, n(%) | 1349 | (32.4%) | 1736 | (40.2%) | 3085 | (36.3%) |
| 5-9, n(%) | 1600 | (38.4%) | 1584 | (36.7%) | 3184 | (37.5%) |
| 10-20, n(%) | 723 | (17.4%) | 655 | (15.2%) | 1378 | (16.2%) |
| Missing, n(%) | 496 | (11.9%) | 343 | (8.0%) | 839 | (9.9%) |
| **GAD-7 score for anxiety – mean (SD)** | 5.9 | (5.6) | 5.2 | (5.4) | 5.6 | (5.5) |
| **Anxiety levels** |  |  |  |  |  |  |
| No anxiety, n(%) | 2032 | (48.8%) | 2321 | (53.8%) | 4353 | (51.3%) |
| Mild anxiety, n(%) | 1047 | (25.1%) | 1050 | (24.3%) | 2097 | (24.7%) |
| Moderate to severe anxiety, n(%) | 952 | (22.8%) | 781 | (18.1%) | 1733 | (20.4%) |
| Missing, n(%) | 137 | (3.3%) | 166 | (3.8%) | 303 | (3.6%) |
| **PHQ-9 score for depression – mean (SD)** | 7.5 | (6.3) | 6.3 | (6.0) | 6.9 | (6.2) |
| **Depression levels** |  |  |  |  |  |  |
| No depression, n(%) | 1663 | (39.9%) | 2078 | (48.1%) | 3741 | (44.1%) |
| Mild to moderate depression, n(%) | 1074 | (25.8%) | 1058 | (24.5%) | 2132 | (25.1%) |
| Moderately severe depression, n(%) | 1289 | (30.9%) | 1017 | (23.6%) | 2306 | (27.2%) |
| Missing, n(%) | 142 | (3.4%) | 165 | (3.8%) | 307 | (3.6%) |

Continuous characteristics summarised across participants with nonmissing data.

IBD: Inflammatory bowel disease; IMD: Indices of Multiple Deprivation; SD: Standard Deviation; BMI: Body Mass Index; EQ-5D-5L: 5-level EQ-5D version.

## Supplementary Table S5: Quality of life (EQ-5D utility) associated with IBD patients’ socio-demographic and clinical characteristics (Initial QoL model)

| **Characteristic** | **Mean (SE)** |
| --- | --- |
| **QoL of reference individual^1^** | 0.862 (0.011)* |
| **Female sex** | -0.037 (0.004)* |
| **Age at time of EQ-5D questionnaire (per 10 years, centred at 50)** | 0.009 (0.002)* |
| **Time since diagnosis (ref: 0-3 years)** |  |
| 4 years and over | 0.023 (0.006)* |
| **Pregnant** | 0.045 (0.026) |
| **Smoking status (ref: never smoker)** |  |
| Ex-smoker | -0.028 (0.005)* |
| Current smoker | -0.059 (0.009)* |
| **BMI (ref: normal weight)** |  |
| Underweight | -0.054 (0.014)* |
| Overweight | -0.038 (0.005)* |
| **IMD quintile (ref: 3)** |  |
| 1 | -0.011 (0.008) |
| 2 | 0.009 (0.007) |
| 4 | -0.002 (0.007) |
| 5 | 0.004 (0.007) |
| **Education level (ref: GCSE or AS/A-levels)** |  |
| No education | -0.030 (0.014)* |
| Further and Higher education | 0.019 (0.005)* |
| **Employment status (ref: employed)** |  |
| Unemployed due to illness | -0.321 (0.010)* |
| Unemployed | -0.106 (0.014)* |
| Student or Homemaker | -0.018 (0.009) |
| Retired | -0.022 (0.007)* |
| **Living circumstances (ref: living alone)** |  |
| Living with someone | 0.026 (0.005)* |
| **IBD type (ref: Crohn's Disease)** |  |
| Other IBD | 0.006 (0.005) |
| **IBD operation** | -0.020 (0.005)* |
| **Use of biologic medication** | -0.021 (0.005)* |
| **Physical comorbidity** | -0.048 (0.004)* |
| **Mental comorbidity** | -0.143 (0.005)* |

IBD activity: Crohn’s Disease activity tertiles (1:0-13, 2:14-94, 3:95 and over), Ulcerative Colitis activity tertiles (1:0, 2:1, 3:2-6); *p-value<0.05

^1^50 years old man with Crohn’s Disease, diagnosed in last 3 years, not pregnant, without operation and not receiving biologic medication, never smoker, normal weight, living alone and in an area of average socioeconomic deprivation, educated at GCSE or AS/A levels, employed and without physical or mental health comorbidities.

## Supplementary Table S6: Quality of life (EQ-5D utility) associated with IBD patients’ socio-demographic, clinical characteristics and severity of symptoms of pain, fatigue and bowel incontinence (N=8486 participants)

|  | **Stage 1** |
| --- | --- |
| **Characteristic** | **Mean (SE)** |
| QoL of reference individual^1^ | 0.961 (0.009)* |
| **Female sex** | 0.004 (0.004) |
| **Age at time of EQ-5D questionnaire (per 10 y, centred at 50)** | 0.003 (0.002) |
| **Time since diagnosis (ref: 0-3 years)** |  |
| 4 years and over | 0.000 (0.005) |
| **Pregnant** | 0.011 (0.022) |
| **Smoking status (ref: never smoker)** |  |
| Ex-smoker | -0.010 (0.004)* |
| Current smoker | -0.023 (0.007)* |
| **BMI (ref: normal weight)** |  |
| Underweight | -0.027 (0.011)* |
| Overweight | -0.017 (0.004)* |
| **IMD quintile (ref: 3)** |  |
| 1 | -0.011 (0.007) |
| 2 | 0.003 (0.006) |
| 4 | -0.003 (0.006) |
| 5 | -0.002 (0.006) |
| **Education level (ref: GCSE or AS/A-levels)** |  |
| No education | -0.013 (0.011) |
| Further and Higher education | 0.002 (0.004) |
| **Employment status (ref: employed)** |  |
| Unemployed due to illness | -0.208 (0.008)* |
| Unemployed | -0.062 (0.011)* |
| Student or Homemaker | -0.015 (0.008) |
| Retired | -0.037 (0.006)* |
| **Living circumstances (ref: living alone)** |  |
| Living with someone | 0.016 (0.004)* |
| **IBD type (ref: Crohn’s Disease)** |  |
| Other IBD | -0.004 (0.004) |
| **IBD operation** | -0.002 (0.004) |
| **Use of biologic medication (ref: No)** | -0.001 (0.004) |
| **Physical comorbidity** | -0.028 (0.004)* |
| **Mental comorbidity** | -0.086 (0.004)* |
| **PROMIS pain score (ref: 0-49, low pain)**** |  |
| 50-59 | -0.071 (0.004)* |
| 60 and over | -0.184 (0.006)* |
| **PROMIS fatigue score (ref: 0-49, low fatigue)**** |  |
| 50-59 | -0.048 (0.004)* |
| 60 and over | -0.161 (0.006)* |
| **PROMIS bowel incontinence score (ref: 0-4, low incontinence)**** |  |
| 5-9 | -0.019 (0.004) |
| 10-20 | -0.063 (0.006)* |

*p-value<0.05; **p_trend_<0.001

^1^50 years old man with Crohn’s Disease, diagnosed in last 3 years, not pregnant, normal weight, living alone and in an area of average socioeconomic deprivation, educated at GCSE or AS/A levels, employed, without operation and not receiving biologic medication, never smoker, without physical or mental health comorbidities, without symptoms of pain, fatigue, bowel incontinence.

IBD: Inflammatory bowel disease; IMD: Indices of Multiple Deprivation; SE: Standard Error; BMI: Body Mass Index; QoL: Quality of Life; PROMIS: Patient-Reported Outcomes Measurement Information System; GAD-7: Generalised Anxiety Disorder Assessment; PHQ-9: Patient Health Questionnaire**.**

## Supplementary Table S7: Quality of life (EQ-5D utility) associated with IBD patients’ socio-demographic, clinical characteristics, symptoms of pain, fatigue and bowel incontinence and interactions with anxiety and depression (N=8486 participants)

| **Characteristic** | **Mean (SE)** |
| --- | --- |
| **QoL of reference individual^1^** | 0.959 (0.009)* |
| **Female sex** | -0.005 (0.004) |
| **Age at time of EQ-5D questionnaire (per 10 y, centred at 50)** | -0.001 (0.002) |
| **Time since diagnosis (ref: 0-3 years)** |  |
| 4 years and over | -0.003 (0.005) |
| **Pregnant** | -0.002 (0.021) |
| **Smoking status (ref: Never smoker)** |  |
| Ex-smoker | -0.013 (0.004)* |
| Current smoker | -0.017 (0.007)* |
| **BMI (ref: normal weight)** |  |
| Underweight | -0.030 (0.011)* |
| Overweight | -0.016 (0.004)* |
| **IMD quintile (ref: 3)** |  |
| 1 | -0.009 (0.006) |
| 2 | 0.001 (0.006) |
| 4 | -0.001 (0.006) |
| 5 | 0.002 (0.005) |
| **Education level (ref: GCSE or AS/A-levels)** |  |
| No education | -0.009 (0.011) |
| Further and Higher education | 0.002 (0.004) |
| **Employment status (ref: employed)** |  |
| Unemployed due to illness | -0.199 (0.008)* |
| Unemployed | -0.055 (0.011)* |
| Student or Homemaker | -0.016 (0.007)* |
| Retired | -0.038 (0.005)* |
| **Living circumstances (ref: living alone)** |  |
| Living with someone | 0.012 (0.004)* |
| **IBD type (ref: Crohn's Disease)** |  |
| Other IBD | 0.000 (0.004) |
| **IBD operation** | -0.005 (0.004) |
| **Use of biologic medication** | -0.003 (0.004) |
| **Physical comorbidity** | -0.028 (0.004)* |
| **Mental comorbidity** | -0.054 (0.004)* |
| **PROMIS pain (Yes)** | -0.108 (0.006)* |
| **PROMIS fatigue (Yes)** | -0.051 (0.009)* |
| **PROMIS bowel incontinence (Yes)** | -0.027 (0.004)* |
| **PROMIS pain (Yes) AND PROMIS fatigue (Yes)** | -0.041 (0.009)* |
| **GAD-7 scale for anxiety (ref: no anxiety)** |  |
| Mild anxiety | -0.040 (0.005)* |
| Moderate to severe anxiety | -0.080 (0.008)* |
| **PHQ-9 scale for depression (ref: no depression)** |  |
| Mild to moderate depression | -0.053 (0.005)* |
| Moderately severe depression | -0.114 (0.007)* |
| **GAD_7_n # PROMIS fatigue (Yes)** |  |
| Mild anxiety AND fatigue | 0.002 (0.011) |
| Moderate to severe anxiety AND fatigue | -0.048 (0.012)* |

PROMIS pain (≥ 60); PROMIS fatigue (≥ 60); PROMIS bowel incontinence (≥ 5); GAD-7 for anxiety (0-4: no or mild anxiety; 5-9 moderate anxiety; 10-20 severe anxiety); PHQ-9 for depression (0-4: no or mild depression; 5-9 moderate depression; 10-20 moderately severe/severe depression); *p-value<0.05; Interactions between anxiety and pain, anxiety and bowel incontinence and, anxiety and depression are not presented as not statistically significant

^1^50 years old man with Crohn’s Disease, diagnosed in last 3 years, not pregnant, normal weight, living alone and in an area of average socioeconomic deprivation, educated at GCSE or AS/A levels, employed, without operation and not receiving biologic medication, never smoker, without physical or mental health comorbidities, without symptoms of pain, fatigue, bowel incontinence, anxiety and depression.

IBD: Inflammatory bowel disease; IMD: Indices of Multiple Deprivation; SE: Standard Error; BMI: Body Mass Index; QoL: Quality of Life; PROMIS: Patient-Reported Outcomes Measurement Information System; GAD-7: Generalised Anxiety Disorder Assessment; PHQ-9: Patient Health Questionnaire.

## Supplementary Table S8: Quality of life (EQ-5D utility) associated with IBD patients’ socio-demographic, clinical characteristics, symptoms of pain, fatigue and bowel incontinence, anxiety and depressions, including adjustments for IBD activity and control (N=8486)

|  | **Stage 1** | **Stage 2** | **Stage 3** |
| --- | --- | --- | --- |
| **Characteristic** | **Mean (SE)** | **Mean (SE)** | **Mean (SE)** |
| QoL of reference individual^1^ | 0.971 (0.009)* | 0.972 (0.009)* | 0.983 (0.009)* |
| Female sex | 0.001 (0.004) | 0.001 (0.004) | 0.000 (0.003) |
| Age at time of EQ-5D questionnaire (per 10 years, centred at 50) | 0.000 (0.002) | 0.000 (0.002) | -0.004 (0.002)* |
| Time since diagnosis (ref: 0-3 years) |  |  |  |
| 4 years and over | -0.007 (0.005) | -0.007 (0.005) | -0.010 (0.005) |
| Pregnant | 0.006 (0.022) | 0.006 (0.022) | -0.003 (0.021) |
| Smoking status (ref: never smoker) |  |  |  |
| Ex-smoker | -0.011 (0.004)* | -0.012 (0.004)* | -0.011 (0.004)* |
| Current smoker | -0.024 (0.007) | -0.024 (0.007)* | -0.017 (0.007)* |
| BMI (ref: normal weight) |  |  |  |
| Underweight | -0.033 (0.011)* | -0.032 (0.011)* | -0.028 (0.011)* |
| Overweight | -0.019 (0.004)* | -0.019 (0.004)* | -0.017 (0.004)* |
| IMD quintile (ref: 3) |  |  |  |
| 1 | -0.009 (0.007) | -0.009 (0.007) | -0.007 (0.006) |
| 2 | 0.004 (0.006) | 0.003 (0.006) | 0.002 (0.006) |
| 4 | -0.002 (0.006) | -0.002 (0.006) | -0.001 (0.005) |
| 5 | -0.001 (0.006) | -0.001 (0.005) | 0.001 (0.005) |
| Education level (ref: GCSE or AS/A-levels) |  |  |  |
| No education | -0.013 (0.011) | -0.013 (0.011) | -0.007 (0.011) |
| Further and Higher education | 0.002 (0.004) | 0.002 (0.004) | 0.001 (0.004) |
| Employment status (ref: employed) |  |  |  |
| Unemployed due to illness | -0.210 (0.008)* | -0.207 (0.008)* | -0.197 (0.008)* |
| Unemployed | -0.063 (0.011)* | -0.062 (0.011)* | -0.055 (0.010)* |
| Student or Homemaker | -0.019 (0.008)* | -0.019 (0.008)* | -0.018 (0.007)* |
| Retired | -0.035 (0.006)* | -0.035 (0.006)* | -0.038 (0.005)* |
| Living circumstances (ref: living alone) |  |  |  |
| Living with someone | 0.016 (0.004) | 0.017 (0.004) | 0.013 (0.004) |
| IBD type (ref: Crohn's Disease) |  |  |  |
| Other IBD | 0.000 (0.004) | -0.001 (0.004) | 0.000 (0.004) |
| IBD operation | 0.008 (0.005) | 0.008 (0.005) | 0.004 (0.005) |
| Use of biologic medication | -0.006 (0.004) | -0.006 (0.004) | -0.004 (0.004) |
| IBD activity (ref: 1, low tertile) |  |  |  |
| 2 | -0.016 (0.005)* | -0.017 (0.005)* | -0.015 (0.004)* |
| 3 (high tertile) | -0.029 (0.005)* | -0.029 (0.005)* | -0.027 (0.005)* |
| 4 (stoma/pouch) | -0.037 (0.008)* | -0.037 (0.008)* | -0.034 (0.008)* |
| IBD control (ref: 1, best control) |  |  |  |
| 2 | -0.057 (0.005)* | -0.060 (0.005)* | -0.039 (0.005)* |
| 3 (worst control) | -0.137 (0.006)* | -0.141 (0.006)* | -0.089 (0.006)* |
| Physical comorbidity | -0.027 (0.004)* | -0.027 (0.004)* | -0.026 (0.003)* |
| Mental comorbidity | -0.085 (0.004)* | -0.084 (0.004)* | -0.054 (0.004)* |
| PROMIS pain | -0.119 (0.005)* | -0.092 (0.007)* | -0.078 (0.006)* |
| PROMIS fatigue | -0.111 (0.005)* | -0.092 (0.006)* | -0.051 (0.006)* |
| PROMIS bowel incontinence | -0.010 (0.004)* | -0.010 (0.004)* | -0.004 (0.004) |
| PROMIS pain AND PROMIS fatigue |  | -0.057 (0.009)* | -0.060 (0.009)* |
| GAD-7 scale for anxiety (ref: no anxiety) |  |  |  |
| Mild anxiety |  |  | -0.032 (0.005)* |
| Moderate to severe anxiety |  |  | -0.094 (0.006)* |
| PHQ-9 scale for depression (ref: no depression) |  |  |  |
| Mild to moderate depression |  |  | -0.033 (0.005)* |
| Moderately severe depression |  |  | -0.085 (0.007)* |

IBD activity: Crohn’s Disease activity tertiles (1:0-13, 2:14-94, 3:95 and over), UC activity tertiles (1:0, 2:1, 3:2-6); IBD control: tertiles (1: 15-16 [best control], 2: 9-14 and 3: 0-8 [worst control]), PROMIS pain (≥ 60); PROMIS fatigue (≥ 60); PROMIS bowel incontinence (≥ 5); *p-value<0.05.

^1^50 years old man with Crohn’s Disease, diagnosed in last 3 years, not pregnant, normal weight, living alone and in an area of average socioeconomic deprivation, educated at GCSE or AS/A levels, employed, without operation and not receiving biologic medication, never smoker, low tertile of IBD activity, best tertile for IBD control, without physical or mental health comorbidities, and in models 2 and 3, without respective symptoms of pain, fatigue, bowel incontinence, anxiety and depression.

## Supplementary Table S9: Quality of life (EQ-5D utility) associated with socio-demographic, clinical characteristics, symptoms of pain, fatigue and bowel incontinence and with anxiety and depression, by IBD type

|  | **Crohn’s Disease (N=4168 participants)** | | | **Other IBD (N=4318 participants)** | | |
| --- | --- | --- | --- | --- | --- | --- |
|  | **Stage 1** | **Stage 2** | **Stage 3** | **Stage 1** | **Stage 2** | **Stage 3** |
| **Characteristic** | **Mean (SE)** | **Mean (SE)** | **Mean (SE)** | **Mean (SE)** | **Mean (SE)** | **Mean (SE)** |
| QoL of reference individual^1^ | 0.931 (0.013)* | 0.929 (0.013)* | 0.963 (0.013)* | 0.919 (0.012)* | 0.919 (0.012)* | 0.952 (0.011)* |
| Female sex | -0.015 (0.006)* | -0.015 (0.006)* | -0.014 (0.005)* | 0.000 (0.005) | 0.000 (0.005) | 0.004 (0.005) |
| Age at time of EQ-5D questionnaire (per 10 years, centred at 50) | 0.006 (0.003)* | 0.006 (0.003)* | -0.002 (0.002) | 0.006 (0.002)* | 0.006 (0.002)* | 0.000 (0.002) |
| Time since diagnosis (ref: 0-3 years) |  |  |  |  |  |  |
| 4 years and over | 0.012 (0.008) | 0.012 (0.008) | 0.004 (0.008) | -0.002 (0.007) | -0.002 (0.007) | -0.009 (0.006) |
| Pregnant | 0.015 (0.030) | 0.015 (0.030) | 0.002 (0.027) | 0.013 (0.033) | 0.013 (0.033) | -0.004 (0.031) |
| Smoking status (ref: never smoker) |  |  |  |  |  |  |
| Ex-smoker | -0.013 (0.006)* | -0.014 (0.006)* | -0.010 (0.005) | -0.017 (0.005)* | -0.017 (0.005)* | -0.016 (0.005)* |
| Current smoker | -0.028 (0.010)* | -0.029 (0.009)* | -0.016 (0.009) | -0.027 (0.013)* | -0.027 (0.013)* | -0.020 (0.012) |
| BMI (ref: normal weight) |  |  |  |  |  |  |
| Underweight | -0.033 (0.016)* | -0.032 (0.016)* | -0.031 (0.015)* | -0.039 (0.019)* | -0.039 (0.019) | -0.026 (0.018) |
| Overweight | -0.015 (0.006)* | -0.016 (0.006)* | -0.013 (0.005)* | -0.024 (0.005)* | -0.024 (0.005)* | -0.019 (0.005)* |
| IMD quintile (ref: 3) |  |  |  |  |  |  |
| 1 | -0.010 (0.010) | -0.010 (0.010) | -0.009 (0.009) | -0.012 (0.009) | -0.012 (0.009) | -0.008 (0.008) |
| 2 | -0.003 (0.009) | -0.003 (0.009) | -0.005 (0.008) | 0.010 (0.008) | 0.010 (0.008) | 0.008 (0.007) |
| 4 | 0.001 (0.009) | 0.001 (0.009) | 0.003 (0.008) | -0.006 (0.008) | -0.006 (0.008) | -0.005 (0.007) |
| 5 | 0.001 (0.008) | 0.001 (0.008) | 0.003 (0.007) | 0.001 (0.007) | 0.001 (0.007) | 0.001 (0.007) |
| Education level (ref: GCSE or AS/A-levels) |  |  |  |  |  |  |
| No education | -0.029 (0.018) | -0.029 (0.018) | -0.018 (0.017) | -0.004 (0.015) | -0.004 (0.015) | 0.000 (0.014) |
| Further and Higher education | 0.003 (0.006) | -0.003 (0.006) | -0.004 (0.005) | 0.011 (0.006) | 0.011 (0.006) | 0.009 (0.005) |
| Employment status (ref: employed) |  |  |  |  |  |  |
| Unemployed due to illness | -0.195 (0.011)* | -0.193 (0.011)* | -0.178 (0.010)* | -0.258 (0.013)* | -0.257 (0.013)* | -0.244 (0.013)* |
| Unemployed | -0.075 (0.016)* | -0.072 (0.016)* | -0.062 (0.014)* | -0.059 (0.017)* | -0.059 (0.017)* | -0.049 (0.016)* |
| Student or Homemaker | -0.021 (0.011) | -0.021 (0.011) | -0.022 (0.011)* | -0.009 (0.011) | -0.009 (0.011) | -0.008 (0.011) |
| Retired | -0.041 (0.009)* | -0.041 (0.009)* | -0.046 (0.008)* | -0.028 (0.008)* | -0.028 (0.008)* | -0.033 (0.007)* |
| Living circumstances (ref: living alone) |  |  |  |  |  |  |
| Living with someone | 0.010 (0.006) | 0.011 (0.006) | 0.004 (0.006) | 0.026 (0.006)* | 0.026 (0.006)* | 0.021 (0.005)* |
| IBD operation | -0.001 (0.006) | -0.001 (0.006) | -0.003 (0.005) | -0.009 (0.008) | -0.009 (0.008) | -0.011 (0.008) |
| Use of biologic medication | -0.002 (0.006) | -0.002 (0.006) | -0.001 (0.006) | -0.011 (0.006) | -0.011 (0.005) | -0.006 (0.005) |
| Physical comorbidity | -0.033 (0.006)* | -0.034 (0.006)* | -0.029 (0.005)* | -0.028 (0.005)* | -0.028 (0.005)* | -0.028 (0.005)* |
| Mental comorbidity | -0.104 (0.006)* | -0.104 (0.006)* | -0.063 (0.006)* | -0.086 (0.006)* | -0.086 (0.006)* | -0.046 (0.006)* |
| PROMIS pain | -0.159 (0.007)* | -0.137 (0.010)* | -0.098 (0.009)* | -0.160 (0.007)* | -0.153 (0.010)* | -0.114 (0.009)* |
| PROMIS fatigue | -0.140 (0.007)* | -0.124 (0.008)* | -0.061 (0.008)* | -0.141 (0.007)* | -0.136 (0.008)* | -0.069 (0.008)* |
| PROMIS bowel incontinence | -0.045 (0.006)* | -0.046 (0.006)* | -0.023 (0.006)* | -0.051 (0.005)* | -0.051 (0.005)* | -0.030 (0.005)* |
| PROMIS pain AND PROMIS fatigue |  | -0.046 (0.014)* | -0.060 (0.013)* |  | -0.017 (0.014) | -0.036 (0.014)* |
| GAD-7 scale for anxiety (ref: no anxiety) |  |  |  |  |  |  |
| Mild anxiety |  |  | -0.044 (0.007)* |  |  | -0.031 (0.006)* |
| Moderate to severe anxiety |  |  | -0.108 (0.009)* |  |  | -0.094 (0.009)* |
| PHQ-9 scale for depression (ref: no depression) |  |  |  |  |  |  |
| Mild to moderate depression |  |  | -0.040 (0.007)* |  |  | -0.059 (0.006)* |
| Moderately severe depression |  |  | -0.110 (0.009)* |  |  | -0.112 (0.009)* |

PROMIS pain (≥ 60); PROMIS fatigue (≥ 60); PROMIS bowel incontinence (≥ 5); *p-value<0.05.

^1^50 years old man with Crohn’s Disease or Other IBD, respectively, diagnosed in last 3 years, not pregnant, normal weight, living alone and in an area of average socioeconomic deprivation, educated at GCSE or AS/A levels, employed, without operation and not receiving biologic medication, never smoker, without physical or mental health comorbidities, and in models 2 and 3, without respective symptoms of pain, fatigue, bowel incontinence, anxiety and depression

## Supplementary Table S10: Quality of life (EQ-5D utility) associated with socio-demographic, clinical characteristics, symptoms of pain, fatigue and bowel incontinence and with anxiety and depression, by gender

|  | **Female (N=4888 participants)** | | | **Male (N=3285 participants)** | | |
| --- | --- | --- | --- | --- | --- | --- |
|  | **Stage 1** | **Stage 2** | **Stage 3** | **Stage 1** | **Stage 2** | **Stage 3** |
| **Characteristic** | **Mean (SE)** | **Mean (SE)** | **Mean (SE)** | **Mean (SE)** | **Mean (SE)** | **Mean (SE)** |
| QoL of reference individual^1^ | 0.911 (0.012)* | 0.909 (0.012)* | 0.942 (0.011)* | 0.947 (0.014)* | 0.946 (0.014)* | 0.982 (0.013)* |
| Age at time of EQ-5D questionnaire (per 10 years, centred at 50) | 0.007 (0.002)* | 0.007 (0.002)* | 0.000 (0.002) | 0.003 (0.003) | 0.003 (0.003) | -0.004 (0.003) |
| Time since diagnosis (ref: 0-3 years) |  |  |  |  |  |  |
| 4 years and over | 0.002 (0.007) | 0.002 (0.007) | -0.003 (0.006) | 0.003 (0.008) | 0.003 (0.008) | -0.007 (0.008) |
| Pregnant | 0.013 (0.023) | 0.014 (0.023) | 0.000 (0.022) | - | - | - |
| Smoking status (ref: never smoker) |  |  |  |  |  |  |
| Ex-smoker | -0.010 (0.005) | -0.011 (0.005) | -0.010 (0.005)* | -0.023 (0.006) | -0.023 (0.006) | -0.017 (0.006)* |
| Current smoker | -0.013 (0.010) | -0.013 (0.010) | -0.003 (0.009) | -0.056 (0.013) | -0.056 (0.013) | -0.043 (0.012)* |
| BMI (ref: normal weight) |  |  |  |  |  |  |
| Underweight | -0.033 (0.014)* | -0.032 (0.014) | -0.020 (0.013) | -0.033 (0.021) | -0.033 (0.021) | -0.037 (0.020) |
| Overweight | -0.022 (0.005)* | -0.022 (0.005) | -0.017 (0.005)* | -0.017 (0.006)* | -0.017 (0.006)* | -0.015 (0.005) |
| IMD quintile (ref: 3) |  |  |  |  |  |  |
| 1 | -0.015 (0.009) | -0.016 (0.009) | -0.012 (0.008) | -0.006 (0.009( | -0.006 (0.009( | -0.005 (0.009) |
| 2 | 0.006 (0.008) | 0.006 (0.008) | 0.004 (0.007) | -0.002 (0.009) | -0.002 (0.009) | -0.003 (0.008) |
| 4 | -0.001 (0.008) | -0.001 (0.008) | 0.000 (0.007) | -0.007 (0.008) | -0.007 (0.008) | -0.006 (0.008) |
| 5 | -0.001 (0.008) | -0.001 (0.008) | 0.003 (0.007) | 0.003 (0.009) | 0.003 (0.009) | 0.000 (0.008) |
| Education level (ref: GCSE or AS/A-levels) |  |  |  |  |  |  |
| No education | -0.038 (0.017)* | -0.036 (0.007)* | -0.024 (0.016) | 0.003 (0.015) | 0.002 (0.015) | 0.004 (0.014) |
| Further and Higher education | 0.002 (0.005) | 0.002 (0.005) | 0.001 (0.005) | 0.005 (0.006) | 0.005 (0.006) | 0.003 (0.006) |
| Employment status (ref: employed) |  |  |  |  |  |  |
| Unemployed due to illness | -0.218 (0.011)* | -0.215 (0.010)* | -0.201 (0.010)* | -0.202 (0.014)* | -0.201 (0.014)* | -0.188 (0.013)* |
| Unemployed | -0.084 (0.016)* | -0.083 (0.016)* | -0.066 (0.015)* | -0.047(0.016)* | -0.047 (0.016)* | -0.044 (0.015)* |
| Student or Homemaker | -0.015 (0.009) | -0.015 (0.009) | -0.014 (0.009) | -0.016 (0.017) | -0.016 (0.017) | -0.022 (0.016) |
| Retired | -0.041 (0.008)* | -0.041 (0.008)* | -0.045 (0.007)* | -0.023 (0.008)* | -0.022 (0.009)* | -0.029 (0.008)* |
| Living circumstances (ref: living alone) |  |  |  |  |  |  |
| Living with someone | 0.016 (0.005)* | 0.016 (0.005)* | 0.012 (0.005)* | 0.021 (0.007)* | 0.021 (0.007)* | 0.013 (0.006)* |
| IBD type (ref: Crohn's Disease) |  |  |  |  |  |  |
| Other IBD | 0.010 (0.006) | 0.009 (0.005) | 0.010 (0.005)* | -0.017 (0.006)* | -0.017 (0.006)* | -0.016 (0.006)* |
| IBD operation | 0.002 (0.006) | 0.002 (0.006) | -0.002 (0.006) | -0.010 (0.007) | -0.010 (0.007) | -0.009 (0.006) |
| Use of biologic medication | -0.004 (0.006) | -0.004 (0.006) | -0.001 (0.006) | -0.010 (0.006) | -0.010 (0.006) | -0.006 (0.006) |
| Physical comorbidity | -0.029 (0.005)* | -0.029 (0.005)* | -0.028 (0.005)* | -0.031 (0.006)* | -0.031 (0.006)* | -0.028 (0.005) |
| Mental comorbidity | -0.097 (0.006)* | -0.097 (0.006)* | -0.059 (0.005)* | -0.095 (0.008)* | -0.094 (0.008)* | -0.050 (0.007) |
| PROMIS pain | -0.163 (0.006)* | -0.140 (0.009)* | -0.106 (0.008)* | -0.157 (0.008)* | -0.151 (0.011)* | -0.103 (0.010)* |
| PROMIS fatigue | -0.132 (0.006)* | -0.117 (0,007)* | -0.058 (0.007)* | -0.162 (0.008)* | -0.157 (0.010)* | -0.080 (0.010)* |
| PROMIS bowel incontinence | -0.040 (0.005)* | -0.041 (0.005)* | -0.023 (0.005)* | -0.057 (0.006)* | -0.058 (0.006)* | -0.032 (0.006)* |
| PROMIS pain AND PROMIS fatigue |  | -0.046 (0.012)* | -0.059 (0.011)* |  | -0.016 (0.017) | -0.041 (0.016) |
| GAD-7 scale for anxiety (ref: no anxiety) |  |  |  |  |  |  |
| Mild anxiety |  |  | -0.034 (0.006)* |  |  | -0.044 (0.007)* |
| Moderate to severe anxiety |  |  | -0.101 (0.008)* |  |  | -0.102 (0.010)* |
| PHQ-9 scale for depression (ref: no depression) |  |  |  |  |  |  |
| Mild to moderate depression |  |  | -0.044 (0.006)* |  |  | -0.058 (0.007)* |
| Moderately severe depression |  |  | -0.103 (0.008)* |  |  | -0.121 (0.010)* |

PROMIS pain (≥ 60); PROMIS fatigue (≥ 60); PROMIS bowel incontinence (≥ 5); *p-value<0.05.

^1^50 years old woman or man, respectively, with Crohn’s Disease, diagnosed in last 3 years, not pregnant (if woman), normal weight, living alone and in an area of average socioeconomic deprivation, educated at GCSE or AS/A levels, employed, without operation and not receiving biologic medication, never smoker, without physical or mental health comorbidities, and in models 2 and 3, without respective symptoms of pain, fatigue, bowel incontinence, anxiety and depression
