# Supplementary material for: Impact of fatigue, pain, and bowel incontinence on the quality of life of people living with Inflammatory Bowel Disease: a UK cross-sectional survey
Source: United European Gastroenterol J. Author manuscript; Available in PMC 2024 Nov 25. (PMC7616848; doi:10.1002/ueg2.12668)
Supplement: Supplementary file 1 [file EMS200112-supplement-Supplementary_Materials.pdf]

Impact of fatigue, pain and bowel incontinence on the quality of life of people living with Inflammatory Bowel Disease: a UK cross-sectional survey

Supplementary material

Supplementary Figure S1: Distribution of EQ-5D-5L domains by IBD-related reported symptom 2

Supplementary Table S1: Definitions of variables included for selection in the final statistical model 4

Supplementary Table S2: Model specifications for impact of IBD symptoms on HRQoL 6

Supplementary Table S3: Socio-demographic characteristics of the 8486 IBD-BOOST survey participants 9

Supplementary Table S4: Clinical characteristics of 8486 participants included in the IBD-BOOST survey by IBD diagnosis 11

Supplementary Table S5: Quality of life (EQ-5D utility) associated with IBD patients' socio-demographic and clinical characteristics (Initial QoL model) 13

Supplementary Table S6: Quality of life (EQ-5D utility) associated with IBD patients' socio-demographic, clinical characteristics and severity of symptoms of fatigue, pain and bowel incontinence (N=8486 participants) 14

Supplementary Table S7: Quality of life (EQ-5D utility) associated with IBD patients' socio-demographic, clinical characteristics, symptoms of fatigue, pain and bowel incontinence and interactions with anxiety and depression (N=8486 participants) 16

Supplementary Table S8: Quality of life (EQ-5D utility) associated with IBD patients' socio-demographic, clinical characteristics, symptoms of fatigue, pain and bowel incontinence, anxiety and depressions and IBD activity and control (N=8486)........ 18

Supplementary Table S9: Quality of life (EQ-5D utility) associated with socio-demographic, clinical characteristics, symptoms of fatigue, pain and bowel incontinence and with anxiety and depression, by IBD type 21

Supplementary Table S10: Quality of life (EQ-5D utility) associated with socio-demographic, clinical characteristics, symptoms of fatigue, pain and bowel incontinence and with anxiety and depression, by gender 24

Supplementary Figure S1: Distribution of EQ-5D-5L domains by IBD-related reported symptom

a) EQ-5D domains among IBD participants with pain

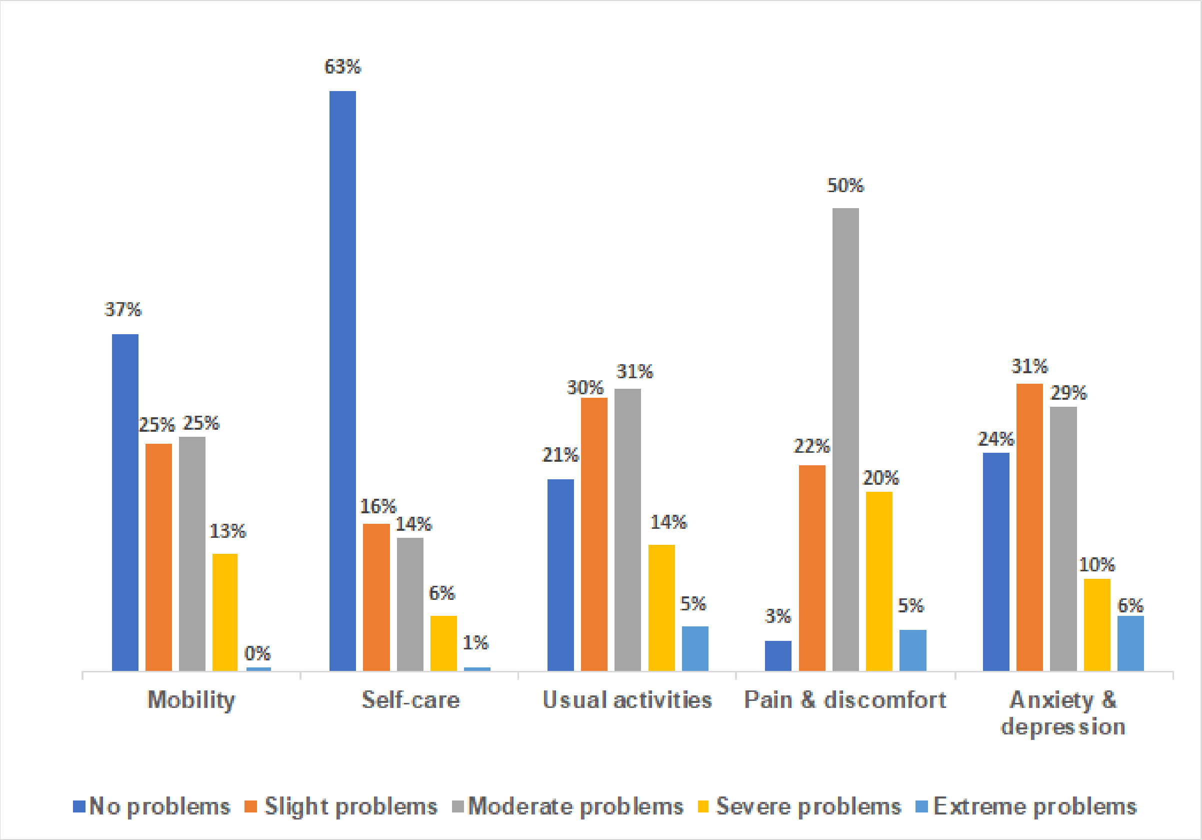

b) EQ-5D domains among IBD participants with fatigue

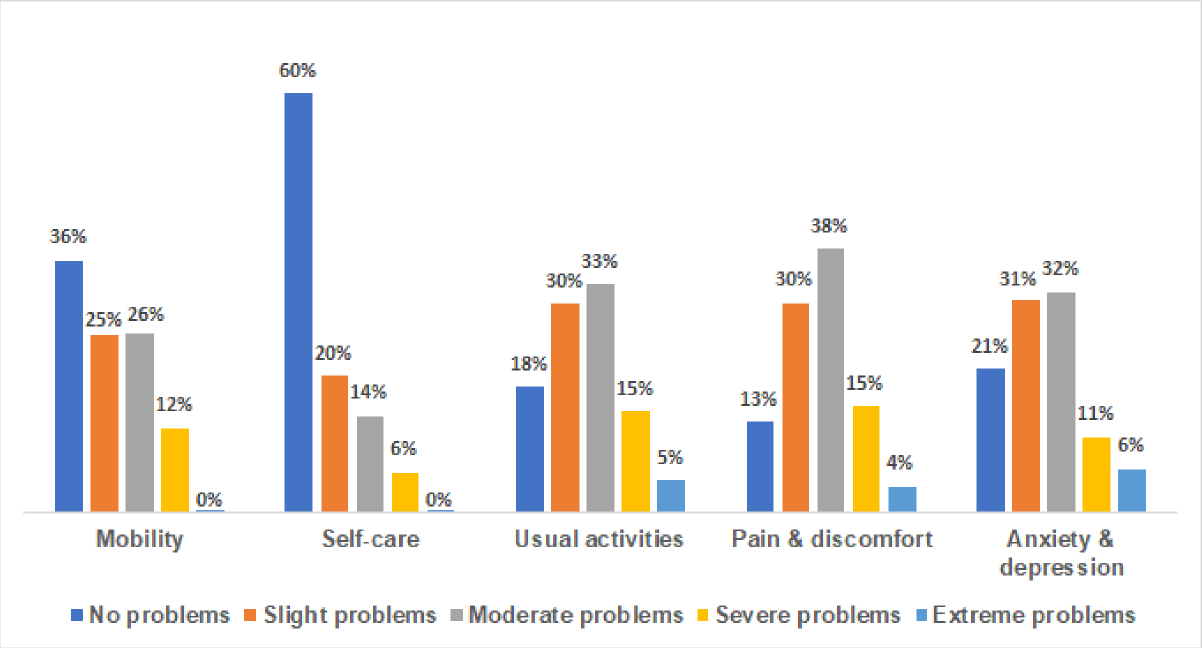

c) EQ-5D domains among IBD participants with incontinence

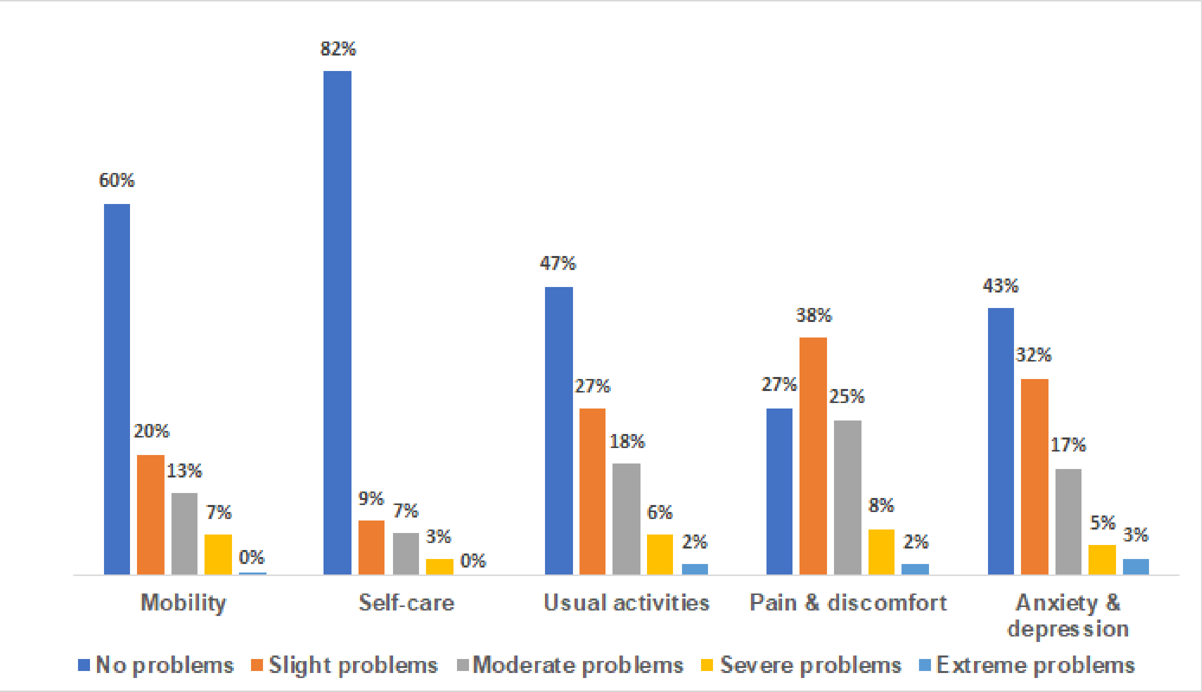

Supplementary Table S1: Definitions of variables included for selection in the final statistical model

Variables	Categories (if categorical)	Note
IBD type	Crohn's disease or Crohn's colitis<br>Other type of IBD	
Age	18-25, 26-35, 36-45, 46-55, 56-65, 66 and over	
Gender	Male, Female	
Smoking status	Never smoked, Ex-smoker, Current smoker	
IMD quintile	1 (most deprived), 2, 3, 4, 5 (least deprived)	Measure of socioeconomic status calculated using self-reported postcode.
BMI (kg/m^2)	<25, >=25-<30, >=30-<35, >=35	Based on self-reported height and weight
IBD surgery in the past	Yes/No	
IBD biologic medication	Yes/No	
IBD activity score or Stoma/pouch	1 (low activity), 2, 3 (high activity), 4 (stoma/pouch)	Tertiles in study population
IBD control score	1 (best control), 2,3 (worst control)	Tertiles in study population
Physical health comorbidity	Yes/No	
Mental health comorbidity	Yes/No	
Time since IBD diagnosis (in years)	0-3, >4	
Currently pregnant	Yes/No	
Education level	No formal education, School (GCSE or AS/A-levels), Further and higher education	
Employment status	Employed, Unemployed due to illness, Unemployed, Student or homemaker, Retired	
Relationship status	Living alone, Living with someone	
Fatigue	0-59 (no fatigue), 60 and over (fatigue)	PROMIS fatigue score (dichotomised)
Pain	0-59 (no pain), 60 and over (pain)	PROMIS pain score (dichotomised)
Incontinence	0-4 (no incontinence), 5-20 (incontinence)	PROMIS Gastrointestinal Bowel Incontinence score (dichotomised)
Anxiety	0-4 (no anxiety), 5-9 (mild anxiety), 10-21 (moderate to severe anxiety)	GAD-7 scale for anxiety
Depression	0-4 (no depression), 5-9 (mild to moderate depression), 10-27(moderately severe depression)	PHQ-9 scale for depression

Abbreviations: IBD: Inflammatory bowel disease; IMD: Indices of Multiple Deprivation; BMI: Body Mass Index; PROMIS: Patient-Reported Outcomes Measurement Information System; GAD-7: Generalised Anxiety Disorder Assessment; PHQ-9: Patient Health Questionnaire.

Supplementary Table S2: Model specifications for impact of IBD symptoms on HRQoL

Outcome	Model	Key exposures	Further predictors	Confounders:
EQ-5D-5L utility	Model stage 1	• PROMIS pain intensity score[1]<br>• PROMIS fatigue score[1]<br>• PROMIS bowel incontinence score[1]	Core (retained in all models)<br>• IBD Type (Crohn's vs other IBD)<br>• Had IBD surgery in past (yes, no)<br>• IBD biologic medication (yes/no)<br><br>Retain only if statistically significant ($p<0.05$):<br>• Time since diagnosis (0-3 years vs 4+ years)	Core (retained in all models)<br>• Age (years) [1]<br>• Gender<br>• Smoking status (never, ex, current)<br>• BMI (underweight, normal, overweight)<br>• IMD quintile (1: least deprived to 5: most deprived)<br>• Physical health comorbidity (yes/no)<br>• Mental health comorbidity (yes/no)<br><br>Enter one at a time in this order and retain only if statistically significant ($p<0.05$):<br>• Currently pregnant (yes/no)<br>• Education (no formal education, school (GCSE or AS/A-levels), "further & higher education")<br>• Employment (paid employment, unemployed due to illness/disease, all other)<br>• Relationship status (in relationship vs not)
EQ-5D-5L utility	Model stage 2	Continuous or categorical (as indicated in Model 1):<br>• PROMIS pain intensity<br>• PROMIS fatigue	• IBD Type (Crohn's vs other IBD)<br>• Had IBD surgery in past (yes, no)	Core (retained in all models)<br>• Age (years)*<br>• Gender<br>• Smoking status (never, ex, current)

		• PROMIS bowel incontinence<br><br>Add all interaction terms between symptoms where more than 20% overlap in symptoms present.<br>Retain interactions only if statistically significant (p<0.05).	• IBD biologic medication (yes/no)<br>• (if statistically significant) Time since diagnosis (0-3 years vs 4+ years)	• BMI (underweight, normal, overweight)<br>• IMD quintile (1: least deprived to 5: most deprived)<br>• Physical health comorbidity (yes/no)<br>• Mental health comorbidity (yes/no)<br><br>Those retained from model stage 1 only from:<br>• Currently pregnant (yes/no)<br>• Education (no formal education, school (GCSE or AS/A-levels), “further & higher education”)<br>• Employment (paid employment, unemployed due to illness/disease, all other)<br>• Relationship status (in relationship vs not)
EQ-5D-5L utility	Model stage 3	Continuous or categorical (as indicated in Model 1):<br>• PROMIS pain intensity<br>• PROMIS fatigue<br>• PROMIS bowel incontinence<br><br>Retained interactions between pain, fatigue, and bowel incontinence.<br><br>Further symptoms:	• IBD Type (Crohn’s vs other IBD)<br>• Had IBD surgery in past (yes, no)<br>• IBD biologic medication (yes/no)<br>• (if statistically significant) Time since diagnosis (0-3 years vs 4+ years)	Core (retained in all models)<br>• Age (years)*<br>• Gender<br>• Smoking status (never, ex, current)<br>• BMI (underweight, normal, overweight)<br>• IMD quintile (1: least deprived to 5: most deprived)<br>• Physical health comorbidity (yes/no)<br>• Mental health comorbidity (yes/no)

		• GAD-7 score for anxiety (no anxiety; mild; moderate to severe anxiety)<br>• PHQ-9 score for depression (no depression; mild to moderate; moderately severe depression)<br><br>Add all interaction terms between the three key symptoms and anxiety and depression where more than 20% overlap symptoms present. Retain interactions only if statistically significant (p<0.05).		Those retained from model stage 1 only from:<br>• Currently pregnant (yes/no)<br>• Education (no formal education, school (GCSE or AS/A-levels), “further & higher education”)<br>• Employment (paid employment, unemployed due to illness/disease, all other)<br>• Relationship status (in relationship vs not)

[1]Nonlinearility in continuous variables will be checked in final models and, if necessary, addressed using categorisation (symptom scores) or spline functions (age)

IBD: Inflammatory bowel disease; IMD: Indices of Multiple Deprivation; BMI: Body Mass Index; EQ-5D-5L: 5-level EQ-5D version; PROMIS: Patient-Reported Outcomes Measurement Information System; GAD-7: Generalised Anxiety Disorder Assessment; PHQ-9: Patient Health Questionnaire.

Supplementary Table S3: Socio-demographic characteristics of the 8486 IBD-BOOST survey participants

Characteristic	Type of IBD					
	Crohn's Disease (n=4168)		Other IBD (n=4318)		Total (n=8486)	
**Gender**						
Male	1479	(35.5%)	1806	(41.8%)	3285	(38.7%)
Female	2550	(61.2%)	2338	(54.1%)	4888	(57.6%)
Missing	139	(3.3%)	174	(4.0%)	313	(3.7%)
**Participant age (in years) mean (SD)**	48.1	(15.1)	51.4	(15.6)	49.8	(15.4)
**Age group**						
18-25	294	(7.1%)	201	(4.7%)	495	(5.8%)
26-35	688	(16.5%)	606	(14.0%)	1294	(15.2%)
36-45	759	(18.2%)	751	(17.4%)	1510	(17.8%)
46-55	917	(22.0%)	758	(17.6%)	1675	(19.7%)
56-65	803	(19.3%)	924	(21.4%)	1727	(20.4%)
66 over	570	(13.7%)	911	(21.1%)	1481	(17.5%)
Missing	137	(3.3%)	167	(3.9%)	304	(3.6%)
**Ethnicity**						
White	3840	(92.1%)	3911	(90.6%)	7751	(91.3%)
Mixed	60	(1.4%)	58	(1.3%)	118	(1.4%)
Asian	78	(1.9%)	131	(3.0%)	209	(2.5%)
Black	22	(0.5%)	13	(0.3%)	35	(0.4%)
Any other	31	(0.7%)	40	(0.9%)	71	(0.8%)
Missing	137	(3.3%)	165	(3.8%)	302	(3.6%)
**Educational level**						
No formal education	98	(2.4%)	121	(2.8%)	219	(2.6%)
School (GCSE or AS/A-levels)	1166	(28.0%)	1111	(25.7%)	2277	(26.8%)
Further and Higher education	2753	(66.1%)	2908	(67.3%)	5661	(66.7%)
Missing	151	(3.6%)	178	(4.1%)	329	(3.9%)
**Employment status**						
Employed	2540	(60.9%)	2531	(58.6%)	5071	(59.8%)
Unemployed due to illness	330	(7.9%)	169	(3.9%)	499	(5.9%)
Unemployed	119	(2.9%)	99	(2.3%)	218	(2.6%)

Student or Homemaker	258	(6.2%)	209	(4.8%)	467	(5.5%)
Retired	778	(18.7%)	1143	(26.5%)	1921	(22.6%)
Missing	143	(3.4%)	167	(3.9%)	310	(3.7%)
**Relationship status**						
Living alone	1260	(30.2%)	1118	(25.9%)	2378	(28.0%)
Living with someone	2756	(66.1%)	3023	(70.0%)	5779	(68.1%)
Missing	152	(3.6%)	177	(4.1%)	329	(3.9%)
**IMD quintile**						
1 (most deprived)	550	(13.2%)	574	(13.3%)	1124	(13.2%)
2	662	(15.9%)	695	(16.1%)	1357	(16.0%)
3	795	(19.1%)	816	(18.9%)	1611	(19.0%)
4	833	(20.0%)	876	(20.3%)	1709	(20.1%)
5 (least deprived)	859	(20.6%)	897	(20.8%)	1756	(20.7%)
Missing	469	(11.3%)	460	(10.7%)	929	(10.9%)
**Smoking status**						
Never smoked	2072	(49.7%)	2093	(48.5%)	4165	(49.1%)
Ex-smoker	1577	(37.8%)	1887	(43.7%)	3464	(40.8%)
Current smoker	367	(8.8%)	162	(3.8%)	529	(6.2%)
Missing	152	(3.6%)	176	(4.1%)	328	(3.9%)
**Alcohol intake**						
Never	1931	(46.3%)	1556	(36.0%)	3487	(41.1%)
1-14 units	1817	(43.6%)	2179	(50.5%)	3996	(47.1%)
15 or more units	268	(6.4%)	406	(9.4%)	674	(7.9%)
Missing	152	(3.6%)	177	(4.1%)	329	(3.9%)

IBD: Inflammatory bowel disease; IMD: Indices of Multiple Deprivation; SD: Standard Deviation; BMI: Body Mass Index; EQ-5D-5L: 5-level EQ-5D version; PROMIS: Patient-Reported Outcomes Measurement Information System; GAD-7: Generalised Anxiety Disorder Assessment; PHQ-9: Patient Health Questionnaire

Supplementary Table S4: Clinical characteristics of 8486 participants included in the IBD-BOOST survey by IBD diagnosis

Characteristic	Type of IBD					
	Crohn's Disease (n=4168)		Other IBD (n=4318)		Total (n=8486)	
**BMI (kg/m²) – mean (SD)**	26.8	(6.0)	26.9	(5.8)	26.8	(5.9)
**BMI ranges**						
Healthy Weight, n(%)	1625	(39.0%)	1590	(36.8%)	3215	(37.9%)
Underweight, n(%)	121	(2.9%)	74	(1.7%)	195	(2.3%)
Overweight, n(%)	2151	(51.6%)	2321	(53.8%)	4472	(52.7%)
Missing, n(%)	271	(6.5%)	333	(7.7%)	604	(7.1%)
**Operation for IBD in the past**						
No, n(%)	1930	(46.3%)	3805	(88.1%)	5735	(67.6%)
Yes, n(%)	2142	(51.4%)	398	(9.2%)	2540	(29.9%)
Missing, n(%)	96	(2.3%)	115	(2.7%)	211	(2.5%)
**Having stoma**						
No, n(%)	3761	(90.2%)	4087	(94.7%)	7848	(92.5%)
Yes, n(%)	382	(9.2%)	167	(3.9%)	549	(6.5%)
Missing, n(%)	25	(0.6%)	64	(1.5%)	89	(1.0%)
**Having pouch**						
No, n(%)	4061	(97.4%)	4114	(95.3%)	8175	(96.3%)
Yes, n(%)	73	(1.8%)	136	(3.1%)	209	(2.5%)
Missing , n(%)	34	(0.8%)	68	(1.6%)	102	(1.2%)
**Had fistula surgery in the past**						
0 , n(%)	3340	(80.1%)	3837	(88.9%)	7177	(84.6%)
1, n(%)	808	(19.4%)	425	(9.8%)	1233	(14.5%)
Missing, n(%)	20	(0.5%)	56	(1.3%)	76	(0.9%)
**Biologic medications**						
No, n(%)	1667	(40.0%)	2425	(56.2%)	4092	(48.2%)
Yes, n(%)	1925	(46.2%)	983	(22.8%)	2908	(34.3%)
Missing, n(%)	576	(13.8%)	910	(21.1%)	1486	(17.5%)
**Having physical comorbidities**						
No, n(%)	2617	(62.8%)	2621	(60.7%)	5238	(61.7%)
Yes, n(%)	1551	(37.2%)	1697	(39.3%)	3248	(38.3%)
**Having mental health comorbidities**						
No, n(%)	3085	(74.0%)	3444	(79.8%)	6529	(76.9%)
Yes, n(%)	1083	(26.0%)	874	(20.2%)	1957	(23.1%)
**Pregnant**						
No, n(%)	3902	(93.6%)	3994	(92.5%)	7896	(93.0%)
Yes, n(%)	37	(0.9%)	26	(0.6%)	63	(0.7%)
Missing, n(%)	229	(5.5%)	298	(6.9%)	527	(6.2%)

**PROMIS fatigue T-score – mean (SD)**	54.8	(8.8)	52.4	(8.9)	53.6	(9.0)
**Fatigue levels**						
0-49 (mild), n(%)	1144	(27.5%)	1618	(37.5%)	2762	(32.6%)
50-59 (moderate) , n(%)	1653	(39.7%)	1609	(37.3%)	3262	(38.4%)
60 and over (severe) , n(%)	1186	(28.5%)	850	(19.7%)	2036	(24.0%)
Missing, n(%)	185	(4.4%)	241	(5.6%)	426	(5.0%)
**PROMIS pain intensity T-score – mean (SD)**	51.3	(11.3)	48.4	(10.9)	49.8	(11.2)
**Pain levels**						
0-49, n(%)	1785	(42.8%)	2240	(51.9%)	4025	(47.4%)
50-59, n(%)	1252	(30.0%)	1178	(27.3%)	2430	(28.6%)
60 and over, n(%)	1041	(25.0%)	728	(16.7%)	1769	(20.9%)
Missing, n(%)	90	(2.2%)	172	(3.9%)	262	(3.1%)
**PROMIS bowel incontinence (raw) score – mean (SD)**	6.8	(3.5)	6.4	(3.4)	6.6	(3.4)
**Incontinence levels**						
0-4, n(%)	1349	(32.4%)	1736	(40.2%)	3085	(36.3%)
5-9, n(%)	1600	(38.4%)	1584	(36.7%)	3184	(37.5%)
10-20, n(%)	723	(17.4%)	655	(15.2%)	1378	(16.2%)
Missing, n(%)	496	(11.9%)	343	(8.0%)	839	(9.9%)
**GAD-7 score for anxiety – mean (SD)**	5.9	(5.6)	5.2	(5.4)	5.6	(5.5)
**Anxiety levels**						
No anxiety, n(%)	2032	(48.8%)	2321	(53.8%)	4353	(51.3%)
Mild anxiety, n(%)	1047	(25.1%)	1050	(24.3%)	2097	(24.7%)
Moderate to severe anxiety, n(%)	952	(22.8%)	781	(18.1%)	1733	(20.4%)
Missing, n(%)	137	(3.3%)	166	(3.8%)	303	(3.6%)
**PHQ-9 score for depression – mean (SD)**	7.5	(6.3)	6.3	(6.0)	6.9	(6.2)
**Depression levels**						
No depression, n(%)	1663	(39.9%)	2078	(48.1%)	3741	(44.1%)
Mild to moderate depression, n(%)	1074	(25.8%)	1058	(24.5%)	2132	(25.1%)
Moderately severe depression, n(%)	1289	(30.9%)	1017	(23.6%)	2306	(27.2%)
Missing, n(%)	142	(3.4%)	165	(3.8%)	307	(3.6%)

Continuous characteristics summarised across participants with nonmissing data.

IBD: Inflammatory bowel disease; IMD: Indices of Multiple Deprivation; SD: Standard Deviation; BMI: Body Mass Index; EQ-5D-5L: 5-level EQ-5D version.

Supplementary Table S5: Quality of life (EQ-5D utility) associated with IBD patients' socio-demographic and clinical characteristics (Initial QoL model)

Characteristic	Mean (SE)
**QoL of reference individual[1]**	0.862 (0.011)*
**Female sex**	-0.037 (0.004)*
**Age at time of EQ-5D questionnaire (per 10 years, centred at 50)**	0.009 (0.002)*
**Time since diagnosis (ref: 0-3 years)**	
4 years and over	0.023 (0.006)*
**Pregnant**	0.045 (0.026)
**Smoking status (ref: never smoker)**	
Ex-smoker	-0.028 (0.005)*
Current smoker	-0.059 (0.009)*
**BMI (ref: normal weight)**	
Underweight	-0.054 (0.014)*
Overweight	-0.038 (0.005)*
**IMD quintile (ref: 3)**	
1	-0.011 (0.008)
2	0.009 (0.007)
4	-0.002 (0.007)
5	0.004 (0.007)
**Education level (ref: GCSE or AS/A-levels)**	
No education	-0.030 (0.014)*
Further and Higher education	0.019 (0.005)*
**Employment status (ref: employed)**	
Unemployed due to illness	-0.321 (0.010)*
Unemployed	-0.106 (0.014)*
Student or Homemaker	-0.018 (0.009)
Retired	-0.022 (0.007)*
**Living circumstances (ref: living alone)**	
Living with someone	0.026 (0.005)*
**IBD type (ref: Crohn's disease)**	
Other IBD	0.006 (0.005)
**IBD operation**	-0.020 (0.005)*
**Use of biologic medication**	-0.021 (0.005)*
**Physical comorbidity**	-0.048 (0.004)*
**Mental comorbidity**	-0.143 (0.005)*

IBD activity: CD activity tertiles (1:0-13, 2:14-94, 3:95 and over), UC activity tertiles (1:0, 2:1, 3:2-6);
*p-value<0.05
[1]50 years old man with Crohn's disease, diagnosed in last 3 years, not pregnant, without operation and not receiving biologic medication, never smoker, normal weight, living alone and in an area of average socioeconomic deprivation, educated at GCSE or AS/A levels, employed and without physical or mental health comorbidities.

Supplementary Table S6: Quality of life (EQ-5D utility) associated with IBD patients' socio-demographic, clinical characteristics and severity of symptoms of fatigue, pain and bowel incontinence (N=8486 participants)

	**Stage 1**
**Characteristic**	**Mean (SE)**
QoL of reference individual[1]	0.961 (0.009)*
**Female sex**	0.004 (0.004)
**Age at time of EQ-5D questionnaire (per 10 y, centred at 50)**	0.003 (0.002)
**Time since diagnosis (ref: 0-3 years)**	
4 years and over	0.000 (0.005)
**Pregnant**	0.011 (0.022)
**Smoking status (ref: never smoker)**	
Ex-smoker	-0.010 (0.004)*
Current smoker	-0.023 (0.007)*
**BMI (ref: normal weight)**	
Underweight	-0.027 (0.011)*
Overweight	-0.017 (0.004)*
**IMD quintile (ref: 3)**	
1	-0.011 (0.007)
2	0.003 (0.006)
4	-0.003 (0.006)
5	-0.002 (0.006)
**Education level (ref: GCSE or AS/A-levels)**	
No education	-0.013 (0.011)
Further and Higher education	0.002 (0.004)
**Employment status (ref: employed)**	
Unemployed due to illness	-0.208 (0.008)*
Unemployed	-0.062 (0.011)*
Student or Homemaker	-0.015 (0.008)
Retired	-0.037 (0.006)*
**Living circumstances (ref: living alone)**	
Living with someone	0.016 (0.004)*
**IBD type (ref: Crohn's disease)**	
Other IBD	-0.004 (0.004)
**IBD operation**	-0.002 (0.004)
**Use of biologic medication (ref: No)**	-0.001 (0.004)
**Physical comorbidity**	-0.028 (0.004)*
**Mental comorbidity**	-0.086 (0.004)*
**PROMIS pain score (ref: 0-49, low pain)****	
50-59	-0.071 (0.004)*

60 and over	-0.184 (0.006)*
**PROMIS fatigue score (ref: 0-49, low fatigue)****	
50-59	-0.048 (0.004)*
60 and over	-0.161 (0.006)*
**PROMIS incontinence score (ref: 0-4, low incontinence)****	
5-9	-0.019 (0.004)
10-20	-0.063 (0.006)*

*p-value<0.05; **p_{trend}<0.001

[1]50 years old man with Crohn's disease, diagnosed in last 3 years, not pregnant, normal weight, living alone and in an area of average socioeconomic deprivation, educated at GCSE or AS/A levels, employed, without operation and not receiving biologic medication, never smoker, without physical or mental health comorbidities, without symptoms of pain, fatigue, incontinence.

IBD: Inflammatory bowel disease; IMD: Indices of Multiple Deprivation; SE: Standard Error; BMI: Body Mass Index; QoL: Quality of Life; PROMIS: Patient-Reported Outcomes Measurement Information System; GAD-7: Generalised Anxiety Disorder Assessment; PHQ-9: Patient Health Questionnaire.

Supplementary Table S7: Quality of life (EQ-5D utility) associated with IBD patients' socio-demographic, clinical characteristics, symptoms of fatigue, pain and bowel incontinence and interactions with anxiety and depression (N=8486 participants)

**Characteristic**	**Mean (SE)**
**QoL of reference individual[1]**	0.959 (0.009)*
**Female sex**	-0.005 (0.004)
**Age at time of EQ-5D questionnaire (per 10 y, centred at 50)**	-0.001 (0.002)
**Time since diagnosis (ref: 0-3 years)**	
4 years and over	-0.003 (0.005)
**Pregnant**	-0.002 (0.021)
**Smoking status (ref: Never smoker)**	
Ex-smoker	-0.013 (0.004)*
Current smoker	-0.017 (0.007)*
**BMI (ref: normal weight)**	
Underweight	-0.030 (0.011)*
Overweight	-0.016 (0.004)*
**IMD quintile (ref: 3)**	
1	-0.009 (0.006)
2	0.001 (0.006)
4	-0.001 (0.006)
5	0.002 (0.005)
**Education level (ref: GCSE or AS/A-levels)**	
No education	-0.009 (0.011)
Further and Higher education	0.002 (0.004)
**Employment status (ref: employed)**	
Unemployed due to illness	-0.199 (0.008)*
Unemployed	-0.055 (0.011)*
Student or Homemaker	-0.016 (0.007)*
Retired	-0.038 (0.005)*
**Living circumstances (ref: living alone)**	
Living with someone	0.012 (0.004)*
**IBD type (ref: Crohn's disease)**	
Other IBD	0.000 (0.004)
**IBD operation**	-0.005 (0.004)
**Use of biologic medication**	-0.003 (0.004)
**Physical comorbidity**	-0.028 (0.004)*
**Mental comorbidity**	-0.054 (0.004)*
**PROMIS pain (Yes)**	-0.108 (0.006)*
**PROMIS fatigue (Yes)**	-0.051 (0.009)*

**PROMIS incontinence (Yes)**	-0.027 (0.004)*
**PROMIS pain (Yes) AND PROMIS fatigue (Yes)**	-0.041 (0.009)*
**GAD-7 scale for anxiety (ref: no anxiety)**	
Mild anxiety	-0.040 (0.005)*
Moderate to severe anxiety	-0.080 (0.008)*
**PHQ-9 scale for depression (ref: no depression)**	
Mild to moderate depression	-0.053 (0.005)*
Moderately severe depression	-0.114 (0.007)*
**GAD_7_n # PROMIS fatigue (Yes)**	
Mild anxiety AND fatigue	0.002 (0.011)
Moderate to severe anxiety AND fatigue	-0.048 (0.012)*

PROMIS pain (≥ 60); PROMIS fatigue (≥ 60); PROMIS bowel incontinence (≥ 5); GAD-7 for anxiety (0-4: no or mild anxiety; 5-9 moderate anxiety; 10-20 severe anxiety); PHQ-9 for depression (0-4: no or mild depression; 5-9 moderate depression; 10-20 moderately severe/severe depression); *p-value<0.05; Interactions between anxiety and pain, anxiety and incontinence and, anxiety and depression are not presented as not statistically significant

[1]50 years old man with Crohn's disease, diagnosed in last 3 years, not pregnant, normal weight, living alone and in an area of average socioeconomic deprivation, educated at GCSE or AS/A levels, employed, without operation and not receiving biologic medication, never smoker, without physical or mental health comorbidities, without symptoms of pain, fatigue, incontinence, anxiety and depression.
IBD: Inflammatory bowel disease; IMD: Indices of Multiple Deprivation; SE: Standard Error; BMI: Body Mass Index; QoL: Quality of Life; PROMIS: Patient-Reported Outcomes Measurement Information System; GAD-7: Generalised Anxiety Disorder Assessment; PHQ-9: Patient Health Questionnaire.

Supplementary Table S8: Quality of life (EQ-5D utility) associated with IBD patients' socio-demographic, clinical characteristics, symptoms of fatigue, pain and bowel incontinence, anxiety and depressions, including adjustments for IBD activity and control (N=8486)

	Stage 1	Stage 2	Stage 3
**Characteristic**	**Mean (SE)**	**Mean (SE)**	**Mean (SE)**
QoL of reference individual[1]	0.971 (0.009)*	0.972 (0.009)*	0.983 (0.009)*
Female sex	0.001 (0.004)	0.001 (0.004)	0.000 (0.003)
Age at time of EQ-5D questionnaire<br>(per 10 years, centred at 50)	0.000 (0.002)	0.000 (0.002)	-0.004 (0.002)*
Time since diagnosis (ref: 0-3 years)			
4 years and over	-0.007 (0.005)	-0.007 (0.005)	-0.010 (0.005)
Pregnant	0.006 (0.022)	0.006 (0.022)	-0.003 (0.021)
Smoking status (ref: never smoker)			
Ex-smoker	-0.011 (0.004)*	-0.012 (0.004)*	-0.011 (0.004)*
Current smoker	-0.024 (0.007)	-0.024 (0.007)*	-0.017 (0.007)*
BMI (ref: normal weight)			
Underweight	-0.033 (0.011)*	-0.032 (0.011)*	-0.028 (0.011)*
Overweight	-0.019 (0.004)*	-0.019 (0.004)*	-0.017 (0.004)*
IMD quintile (ref: 3)			
1	-0.009 (0.007)	-0.009 (0.007)	-0.007 (0.006)
2	0.004 (0.006)	0.003 (0.006)	0.002 (0.006)
4	-0.002 (0.006)	-0.002 (0.006)	-0.001 (0.005)
5	-0.001 (0.006)	-0.001 (0.005)	0.001 (0.005)
Education level (ref: GCSE or AS/A-			
No education	-0.013 (0.011)	-0.013 (0.011)	-0.007 (0.011)

Further and Higher education	0.002 (0.004)	0.002 (0.004)	0.001 (0.004)
Employment status (ref: employed)			
Unemployed due to illness	-0.210 (0.008)*	-0.207 (0.008)*	-0.197 (0.008)*
Unemployed	-0.063 (0.011)*	-0.062 (0.011)*	-0.055 (0.010)*
Student or Homemaker	-0.019 (0.008)*	-0.019 (0.008)*	-0.018 (0.007)*
Retired	-0.035 (0.006)*	-0.035 (0.006)*	-0.038 (0.005)*
Living circumstances (ref: living alone)			
Living with someone	0.016 (0.004)	0.017 (0.004)	0.013 (0.004)
IBD type (ref: Crohn's disease)			
Other IBD	0.000 (0.004)	-0.001 (0.004)	0.000 (0.004)
IBD operation	0.008 (0.005)	0.008 (0.005)	0.004 (0.005)
Use of biologic medication	-0.006 (0.004)	-0.006 (0.004)	-0.004 (0.004)
IBD activity (ref: 1, low tertile)			
2	-0.016 (0.005)*	-0.017 (0.005)*	-0.015 (0.004)*
3 (high tertile)	-0.029 (0.005)*	-0.029 (0.005)*	-0.027 (0.005)*
4 (stoma/pouch)	-0.037 (0.008)*	-0.037 (0.008)*	-0.034 (0.008)*
IBD control (ref: 1, best control)			
2	-0.057 (0.005)*	-0.060 (0.005)*	-0.039 (0.005)*
3 (worst control)	-0.137 (0.006)*	-0.141 (0.006)*	-0.089 (0.006)*
Physical comorbidity	-0.027 (0.004)*	-0.027 (0.004)*	-0.026 (0.003)*
Mental comorbidity	-0.085 (0.004)*	-0.084 (0.004)*	-0.054 (0.004)*
PROMIS pain	-0.119 (0.005)*	-0.092 (0.007)*	-0.078 (0.006)*
PROMIS fatigue	-0.111 (0.005)*	-0.092 (0.006)*	-0.051 (0.006)*
PROMIS incontinence	-0.010 (0.004)*	-0.010 (0.004)*	-0.004 (0.004)
PROMIS pain AND PROMIS fatigue		-0.057 (0.009)*	-0.060 (0.009)*

GAD-7 scale for anxiety (ref: no anxiety)			
Mild anxiety			-0.032 (0.005)*
Moderate to severe anxiety			-0.094 (0.006)*
PHQ-9 scale for depression (ref: no depression)			
Mild to moderate depression			-0.033 (0.005)*
Moderately severe depression			-0.085 (0.007)*

IBD activity: CD activity tertiles (1:0-13, 2:14-94, 3:95 and over), UC activity tertiles (1:0, 2:1, 3:2-6); IBD control: tertiles (1: 15-16 [best control], 2: 9-14 and 3: 0-8 [worst control]), PROMIS pain (≥ 60); PROMIS fatigue (≥ 60); PROMIS bowel incontinence (≥ 5); *p-value<0.05.

[1]50 years old man with Crohn's disease, diagnosed in last 3 years, not pregnant, normal weight, living alone and in an area of average socioeconomic deprivation, educated at GCSE or AS/A levels, employed, without operation and not receiving biologic medication, never smoker, low tertile of IBD activity, best tertile for IBD control, without physical or mental health comorbidities, and in models 2 and 3, without respective symptoms of pain, fatigue, incontinence, anxiety and depression.

Supplementary Table S9: Quality of life (EQ-5D utility) associated with socio-demographic, clinical characteristics, symptoms of fatigue, pain and bowel incontinence and with anxiety and depression, by IBD type

	CD (N=4168 participants)			Other IBD (N=4318 participants)		
	Stage 1	Stage 2	Stage 3	Stage 1	Stage 2	Stage 3
Characteristic	Mean (SE)	Mean (SE)	Mean (SE)	Mean (SE)	Mean (SE)	Mean (SE)
QoL of reference individual[1]	0.931 (0.013)*	0.929 (0.013)*	0.963 (0.013)*	0.919 (0.012)*	0.919 (0.012)*	0.952 (0.011)*
Female sex	-0.015 (0.006)*	-0.015 (0.006)*	-0.014 (0.005)*	0.000 (0.005)	0.000 (0.005)	0.004 (0.005)
Age at time of EQ-5D questionnaire (per 10 years, centred at 50)	0.006 (0.003)*	0.006 (0.003)*	-0.002 (0.002)	0.006 (0.002)*	0.006 (0.002)*	0.000 (0.002)
Time since diagnosis (ref: 0-3 years)						
4 years and over	0.012 (0.008)	0.012 (0.008)	0.004 (0.008)	-0.002 (0.007)	-0.002 (0.007)	-0.009 (0.006)
Pregnant	0.015 (0.030)	0.015 (0.030)	0.002 (0.027)	0.013 (0.033)	0.013 (0.033)	-0.004 (0.031)
Smoking status (ref: never smoker)						
Ex-smoker	-0.013 (0.006)*	-0.014 (0.006)*	-0.010 (0.005)	-0.017 (0.005)*	-0.017 (0.005)*	-0.016 (0.005)*
Current smoker	-0.028 (0.010)*	-0.029 (0.009)*	-0.016 (0.009)	-0.027 (0.013)*	-0.027 (0.013)*	-0.020 (0.012)
BMI (ref: normal weight)						
Underweight	-0.033 (0.016)*	-0.032 (0.016)*	-0.031 (0.015)*	-0.039 (0.019)*	-0.039 (0.019)	-0.026 (0.018)

Overweight	-0.015 (0.006)*	-0.016 (0.006)*	-0.013 (0.005)*	-0.024 (0.005)*	-0.024 (0.005)*	-0.019 (0.005)*
IMD quintile (ref: 3)						
1	-0.010 (0.010)	-0.010 (0.010)	-0.009 (0.009)	-0.012 (0.009)	-0.012 (0.009)	-0.008 (0.008)
2	-0.003 (0.009)	-0.003 (0.009)	-0.005 (0.008)	0.010 (0.008)	0.010 (0.008)	0.008 (0.007)
4	0.001 (0.009)	0.001 (0.009)	0.003 (0.008)	-0.006 (0.008)	-0.006 (0.008)	-0.005 (0.007)
5	0.001 (0.008)	0.001 (0.008)	0.003 (0.007)	0.001 (0.007)	0.001 (0.007)	0.001 (0.007)
Education level (ref: GCSE or AS/A-						
No education	-0.029 (0.018)	-0.029 (0.018)	-0.018 (0.017)	-0.004 (0.015)	-0.004 (0.015)	0.000 (0.014)
Further and Higher education	0.003 (0.006)	-0.003 (0.006)	-0.004 (0.005)	0.011 (0.006)	0.011 (0.006)	0.009 (0.005)
Employment status (ref: employed)						
Unemployed due to illness	-0.195 (0.011)*	-0.193 (0.011)*	-0.178 (0.010)*	-0.258 (0.013)*	-0.257 (0.013)*	-0.244 (0.013)*
Unemployed	-0.075 (0.016)*	-0.072 (0.016)*	-0.062 (0.014)*	-0.059 (0.017)*	-0.059 (0.017)*	-0.049 (0.016)*
Student or Homemaker	-0.021 (0.011)	-0.021 (0.011)	-0.022 (0.011)*	-0.009 (0.011)	-0.009 (0.011)	-0.008 (0.011)
Retired	-0.041 (0.009)*	-0.041 (0.009)*	-0.046 (0.008)*	-0.028 (0.008)*	-0.028 (0.008)*	-0.033 (0.007)*
Living circumstances (ref: living alone)						
Living with someone	0.010 (0.006)	0.011 (0.006)	0.004 (0.006)	0.026 (0.006)*	0.026 (0.006)*	0.021 (0.005)*
IBD operation	-0.001 (0.006)	-0.001 (0.006)	-0.003 (0.005)	-0.009 (0.008)	-0.009 (0.008)	-0.011 (0.008)

Use of biologic medication	-0.002 (0.006)	-0.002 (0.006)	-0.001 (0.006)	-0.011 (0.006)	-0.011 (0.005)	-0.006 (0.005)
Physical comorbidity	-0.033 (0.006)*	-0.034 (0.006)*	-0.029 (0.005)*	-0.028 (0.005)*	-0.028 (0.005)*	-0.028 (0.005)*
Mental comorbidity	-0.104 (0.006)*	-0.104 (0.006)*	-0.063 (0.006)*	-0.086 (0.006)*	-0.086 (0.006)*	-0.046 (0.006)*
PROMIS pain	-0.159 (0.007)*	-0.137 (0.010)*	-0.098 (0.009)*	-0.160 (0.007)*	-0.153 (0.010)*	-0.114 (0.009)*
PROMIS fatigue	-0.140 (0.007)*	-0.124 (0.008)*	-0.061 (0.008)*	-0.141 (0.007)*	-0.136 (0.008)*	-0.069 (0.008)*
PROMIS incontinence	-0.045 (0.006)*	-0.046 (0.006)*	-0.023 (0.006)*	-0.051 (0.005)*	-0.051 (0.005)*	-0.030 (0.005)*
PROMIS pain AND PROMIS fatigue		-0.046 (0.014)*	-0.060 (0.013)*		-0.017 (0.014)	-0.036 (0.014)*
GAD-7 scale for anxiety (ref: no anxiety)						
Mild anxiety			-0.044 (0.007)*			-0.031 (0.006)*
Moderate to severe anxiety			-0.108 (0.009)*			-0.094 (0.009)*
PHQ-9 scale for depression (ref: no depression)						
Mild to moderate depression			-0.040 (0.007)*			-0.059 (0.006)*
Moderately severe depression			-0.110 (0.009)*			-0.112 (0.009)*

PROMIS pain (≥ 60); PROMIS fatigue (≥ 60); PROMIS bowel incontinence (≥ 5); *p-value<0.05.

[1]50 years old man with Crohn's disease or Other IBD, respectively, diagnosed in last 3 years, not pregnant, normal weight, living alone and in an area of average socioeconomic deprivation, educated at GCSE or AS/A levels, employed, without operation and not receiving biologic medication, never smoker, without physical or mental health comorbidities, and in models 2 and 3, without respective symptoms of pain, fatigue, incontinence, anxiety and depression

Supplementary Table S10: Quality of life (EQ-5D utility) associated with socio-demographic, clinical characteristics, symptoms of fatigue, pain and bowel incontinence and with anxiety and depression, by gender

	Female (N=4888 participants)			Male (N=3285 participants)		
	Stage 1	Stage 2	Stage 3	Stage 1	Stage 2	Stage 3
**Characteristic**	**Mean (SE)**	**Mean (SE)**	**Mean (SE)**	**Mean (SE)**	**Mean (SE)**	**Mean (SE)**
QoL of reference individual[1]	0.911 (0.012)*	0.909 (0.012)*	0.942 (0.011)*	0.947 (0.014)*	0.946 (0.014)*	0.982 (0.013)*
Age at time of EQ-5D questionnaire (per 10 years, centred at 50)	0.007 (0.002)*	0.007 (0.002)*	0.000 (0.002)	0.003 (0.003)	0.003 (0.003)	-0.004 (0.003)
Time since diagnosis (ref: 0-3 years)						
4 years and over	0.002 (0.007)	0.002 (0.007)	-0.003 (0.006)	0.003 (0.008)	0.003 (0.008)	-0.007 (0.008)
Pregnant	0.013 (0.023)	0.014 (0.023)	0.000 (0.022)	-	-	-
Smoking status (ref: never smoker)						
Ex-smoker	-0.010 (0.005)	-0.011 (0.005)	-0.010 (0.005)*	-0.023 (0.006)	-0.023 (0.006)	-0.017 (0.006)*
Current smoker	-0.013 (0.010)	-0.013 (0.010)	-0.003 (0.009)	-0.056 (0.013)	-0.056 (0.013)	-0.043 (0.012)*
BMI (ref: normal weight)						
Underweight	-0.033 (0.014)*	-0.032 (0.014)	-0.020 (0.013)	-0.033 (0.021)	-0.033 (0.021)	-0.037 (0.020)
Overweight	-0.022 (0.005)*	-0.022 (0.005)	-0.017 (0.005)*	-0.017 (0.006)*	-0.017 (0.006)*	-0.015 (0.005)

IMD quintile (ref: 3)						
1	-0.015 (0.009)	-0.016 (0.009)	-0.012 (0.008)	-0.006 (0.009(	-0.006 (0.009(	-0.005 (0.009)
2	0.006 (0.008)	0.006 (0.008)	0.004 (0.007)	-0.002 (0.009)	-0.002 (0.009)	-0.003 (0.008)
4	-0.001 (0.008)	-0.001 (0.008)	0.000 (0.007)	-0.007 (0.008)	-0.007 (0.008)	-0.006 (0.008)
5	-0.001 (0.008)	-0.001 (0.008)	0.003 (0.007)	0.003 (0.009)	0.003 (0.009)	0.000 (0.008)
Education level (ref: GCSE or AS/A-						
No education	-0.038 (0.017)*	-0.036 (0.007)*	-0.024 (0.016)	0.003 (0.015)	0.002 (0.015)	0.004 (0.014)
Further and Higher education	0.002 (0.005)	0.002 (0.005)	0.001 (0.005)	0.005 (0.006)	0.005 (0.006)	0.003 (0.006)
Employment status (ref: employed)						
Unemployed due to illness	-0.218 (0.011)*	-0.215 (0.010)*	-0.201 (0.010)*	-0.202 (0.014)*	-0.201 (0.014)*	-0.188 (0.013)*
Unemployed	-0.084 (0.016)*	-0.083 (0.016)*	-0.066 (0.015)*	-0.047(0.016)*	-0.047 (0.016)*	-0.044 (0.015)*
Student or Homemaker	-0.015 (0.009)	-0.015 (0.009)	-0.014 (0.009)	-0.016 (0.017)	-0.016 (0.017)	-0.022 (0.016)
Retired	-0.041 (0.008)*	-0.041 (0.008)*	-0.045 (0.007)*	-0.023 (0.008)*	-0.022 (0.009)*	-0.029 (0.008)*
Living circumstances (ref: living alone)						
Living with someone	0.016 (0.005)*	0.016 (0.005)*	0.012 (0.005)*	0.021 (0.007)*	0.021 (0.007)*	0.013 (0.006)*
IBD type (ref: Crohn's disease)						
Other IBD	0.010 (0.006)	0.009 (0.005)	0.010 (0.005)*	-0.017 (0.006)*	-0.017 (0.006)*	-0.016 (0.006)*
IBD operation	0.002 (0.006)	0.002 (0.006)	-0.002 (0.006)	-0.010 (0.007)	-0.010 (0.007)	-0.009 (0.006)

Use of biologic medication	-0.004 (0.006)	-0.004 (0.006)	-0.001 (0.006)	-0.010 (0.006)	-0.010 (0.006)	-0.006 (0.006)
Physical comorbidity	-0.029 (0.005)*	-0.029 (0.005)*	-0.028 (0.005)*	-0.031 (0.006)*	-0.031 (0.006)*	-0.028 (0.005)
Mental comorbidity	-0.097 (0.006)*	-0.097 (0.006)*	-0.059 (0.005)*	-0.095 (0.008)*	-0.094 (0.008)*	-0.050 (0.007)
PROMIS pain	-0.163 (0.006)*	-0.140 (0.009)*	-0.106 (0.008)*	-0.157 (0.008)*	-0.151 (0.011)*	-0.103 (0.010)*
PROMIS fatigue	-0.132 (0.006)*	-0.117 (0,007)*	-0.058 (0.007)*	-0.162 (0.008)*	-0.157 (0.010)*	-0.080 (0.010)*
PROMIS incontinence	-0.040 (0.005)*	-0.041 (0.005)*	-0.023 (0.005)*	-0.057 (0.006)*	-0.058 (0.006)*	-0.032 (0.006)*
PROMIS pain AND PROMIS fatigue		-0.046 (0.012)*	-0.059 (0.011)*		-0.016 (0.017)	-0.041 (0.016)
GAD-7 scale for anxiety (ref: no anxiety)						
Mild anxiety			-0.034 (0.006)*			-0.044 (0.007)*
Moderate to severe anxiety			-0.101 (0.008)*			-0.102 (0.010)*
PHQ-9 scale for depression (ref: no depression)						
Mild to moderate depression			-0.044 (0.006)*			-0.058 (0.007)*
Moderately severe depression			-0.103 (0.008)*			-0.121 (0.010)*

PROMIS pain (≥ 60); PROMIS fatigue (≥ 60); PROMIS bowel incontinence (≥ 5); *p-value<0.05.

[1]50 years old woman or man, respectively, with Crohn's disease, diagnosed in last 3 years, not pregnant (if woman), normal weight, living alone and in an area of average socioeconomic deprivation, educated at GCSE or AS/A levels, employed, without operation and not receiving biologic medication, never smoker, without physical or mental health comorbidities, and in models 2 and 3, without respective symptoms of pain, fatigue, incontinence, anxiety and depression
